# Differential reinforcement of cGAS-STING pathway-involved immunotherapy by biomineralized bacterial outer membrane-sensitized EBRT and RNT

**DOI:** 10.1186/s12951-024-02565-7

**Published:** 2024-06-03

**Authors:** Mengling Shen, Li Guo, Hengyu Zhang, Bingshu Zheng, Xinpei Liu, Jingyu Gu, Tao Yang, Chunfeng Sun, Xuan Yi

**Affiliations:** 1 School of Pharmacy, Jiangsu Key Laboratory of Inflammation and Molecular Drug Targets, Nantong University, Nantong, 226001 Jiangsu China; 2 Department of Nuclear Medicine, Affiliated Hospital of Nantong University, Nantong, 226001 Jiangsu China; 3 Department of Radiotherapy, Affiliated Hospital of Nantong University, Nantong, 226001 Jiangsu China

**Keywords:** Bacterial outer membrane, Radiotherapy, cGAS-STING pathway, Hypoxia, Immunotherapy

## Abstract

Radiotherapy (RT), including external beam radiation therapy (EBRT) and radionuclide therapy (RNT), realizes physical killing of local tumors and activates systemic anti-tumor immunity. However, these effects need to be further strengthened and the difference between EBRT and RNT should be discovered. Herein, bacterial outer membrane (OM) was biomineralized with manganese oxide (MnO_2_) to obtain OM@MnO_2_-PEG nanoparticles for enhanced radio-immunotherapy via amplifying EBRT/RNT-induced immunogenic cell death (ICD) and cyclic GMP-AMP synthase (cGAS)-stimulator of interferon genes (STING) activation. OM@MnO_2_-PEG can react with H_2_O_2_ and then gradually produce O_2_, Mn^2+^ and OM fragments in the tumor microenvironment. The relieved tumor hypoxia improves the radio-sensitivity of tumor cells, resulting in enhanced ICD and DNA damage. Mn^2+^ together with the DNA fragments in the cytoplasm activate the cGAS-STING pathway, further exhibiting a positive role in various aspects of innate immunity and adaptive immunity. Besides, OM fragments promote tumor antigen presentation and anti-tumor macrophages polarization. More importantly, our study reveals that OM@MnO_2_-PEG-mediated RNT triggers much stronger cGAS-STING pathway-involved immunotherapy than that of EBRT, owing to the duration difference of RT. Therefore, this study develops a powerful sensitizer of radio-immunotherapy and uncovers some differences between EBRT and RNT in the activation of cGAS-STING pathway-related anti-tumor immunity.

**Graphical Abstract:**

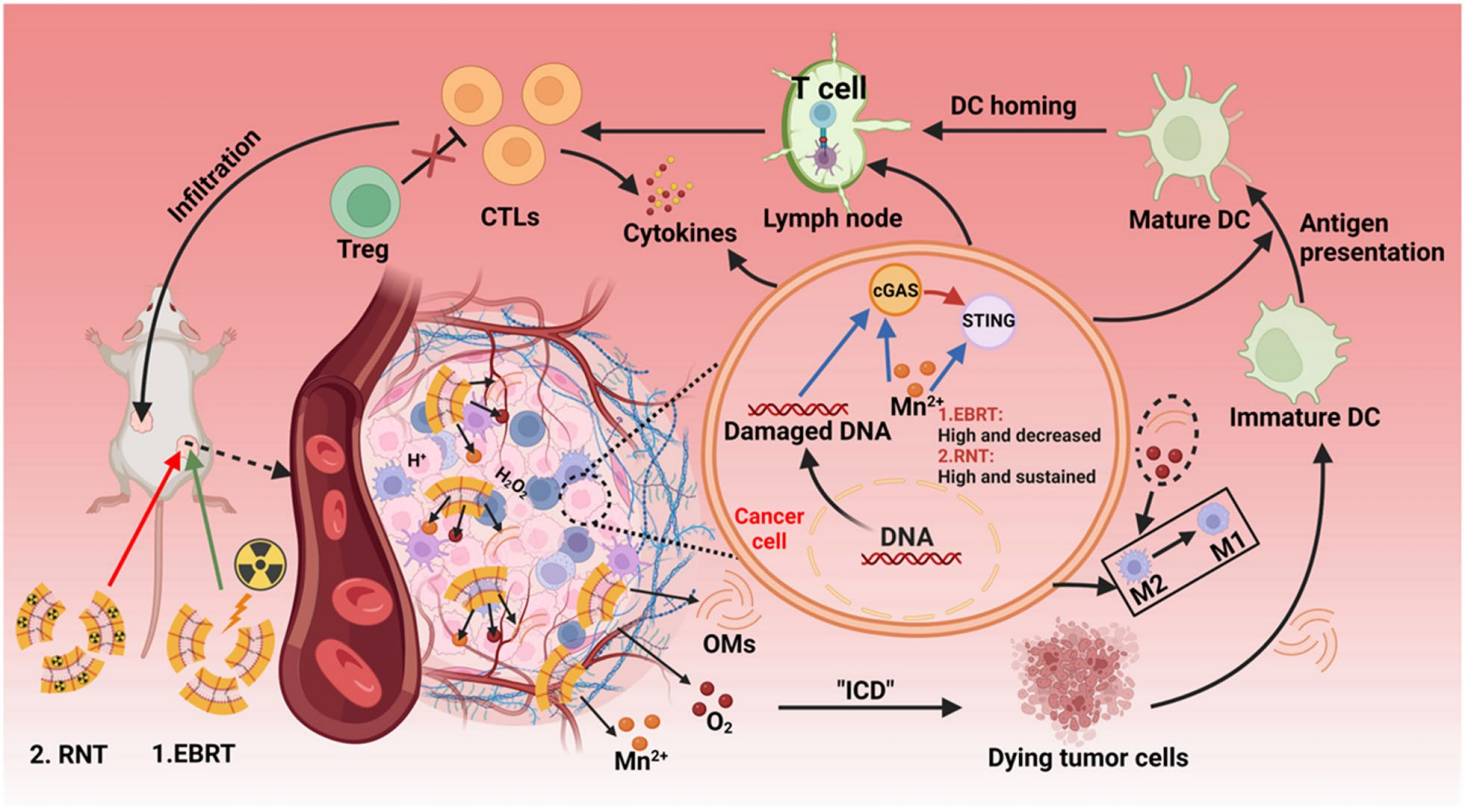

**Supplementary Information:**

The online version contains supplementary material available at 10.1186/s12951-024-02565-7.

## Introduction

Radiotherapy (RT) is a fascinating treatment for most cancer and can be further divided into external beam radiation therapy (EBRT) and radionuclide therapy (RNT) [1, 2]. Notably, RT does not only directly kill the tumor cells by generating various reactive oxygen species, but also triggers the systemic anti-tumor immunity [3, 4]. The infiltration, polarization and efficiency of various immune cells, including innate and adaptive immune cells, have significantly changed after RT treatment [5–7]. RT promotes the immunogenic cell death (ICD) and the release of tumor-specific antigen in tumor tissue, together activating DC cells and CD8^+^ T cells (CTLs) for anti-tumor immunotherapy [8–10]. More importantly, RT-damaged DNA fragments would leak into the cytoplasm from nucleus or mitochondria, further stimulating cyclic GMP-AMP synthase (cGAS)-stimulator of interferon genes (STING) pathway [11, 12]. The cGAS-STING activation shows powerful and positive effect on strengthening the T cells-mediated anti-tumor immunity and reversing M2 macrophage-mediated tumor immunosuppressive microenvironment by secreting type I interferon and various inflammatory cytokines [13–15]. Meanwhile, as the other side of a double-edged sword, RT up-regulates the expression of various immune checkpoint-related proteins, such as PD-L1, CTLA-4 and IDO-1, and decreases the number of lymphocytes in the body by causing severe bone marrow damage, negatively modulating the anti-tumor immunity [16–19]. Nowadays, both of EBRT and RNT, which possess the same way to trigger biological effects, are needed optimized and considered as important treatment to control the local tumor tissue and stimulate the systemic immune response [20–22]. The respective source of ionizing radiation in EBRT and RNT leads to the intermittent/short-term exposure to radiation for EBRT and continuous/long-term exposure to radiation for RNT. Whether this temporal difference can result in differences in immune stimulation between EBRT and RNT has not been adequately studied.

Additionally, in view of the above immune stimulation characteristics of RT, a large number of biomaterials are being developed for enhanced radio-immunotherapy of cancer [23–25]. Generally, most of the radio-sensitizers can promote RT to cause DNA damage, further improving the ICD and cGAS-STING activation for amplifying the immune-stimulatory efficacy of RT [8, 9, 11, 12, 26]. Among them, oxygen-delivery/producing nanoparticles are the representative radio-sensitizing agents via relieving the tumor hypoxia [22, 27–32]. Moreover, the generation of oxygen affects the energy metabolism of immune cells in the tumor and polarizes the tumor-associated macrophage into the anti-tumor M1 type instead of the pro-tumor M2 type [33–35]. Besides, related biomaterials have been selected or designed according to the special immune microenvironment induced by RT to strengthen the each process of the T cells-mediated anti-tumor immunity, including antigen release, antigen presentation, DC maturation, T cell infiltration and CTL effect, for systemic anti-tumor adaptive immunotherapy [19, 36–38]. Notably, bacteria and bacterial outer membrane vesicles (OMVs) can specifically cluster in the tumor area and involve in multiple steps to promote the anti-tumor immunity, mediating perfect combination treatment effect of immunotherapy and RT [39–43]. Additionally, metal ions, including Mn^2+^, Zn^2+^ and Hf^4+^, have been reported to enhance the sensitivity of cGAS to cytoplasmic DNA and trigger the production of the second messenger cGAMP, thus playing a key role in the effective and stable activation of cGAS-STING pathway [12, 44–46]. The existence of these metal ions in the tumor maintains the RT-induced cGAS-STING activation and enlarges the RT-triggered anti-tumor immunity.

**Fig. 1:**
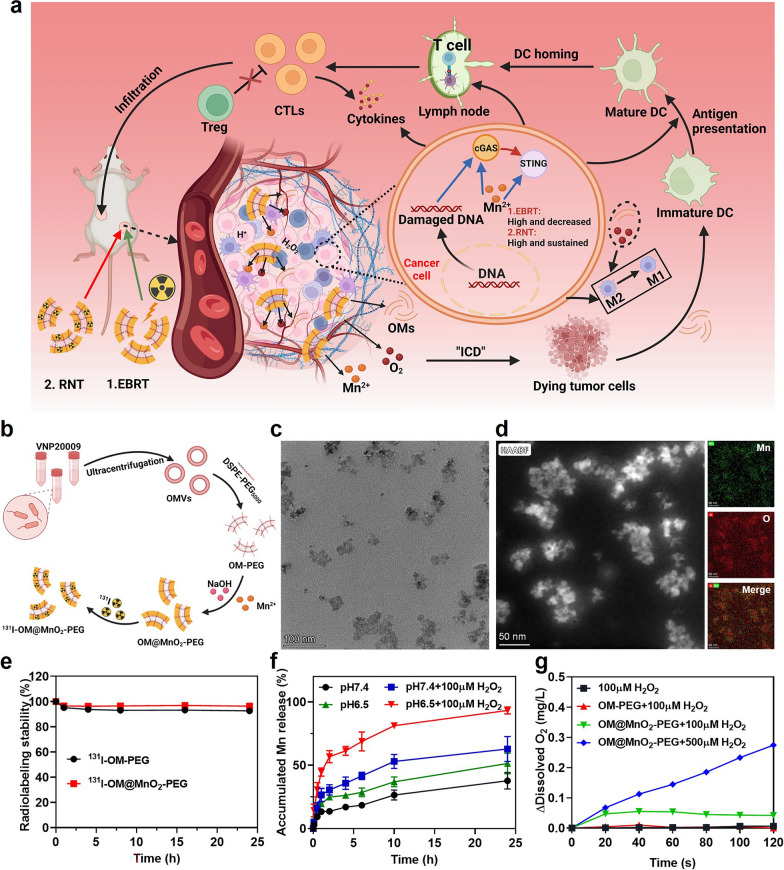
The preparation and characterization of OM@MnO_2_-PEG. **a** The schematic diagram of the enhanced systemic anti-tumor immunity induced by OM@MnO_2_-PEG-mediated RT. **b** Schematic diagram showing the synthesis of ^131^I-OM@MnO_2_-PEG. **c** TEM of OM@MnO_2_-PEG (scale bar 100 nm). **d** Elemental mapping analysis of OM@MnO_2_-PEG (scale bar 50 nm). **e** Radio-labeling stability of radioactive iodine for OM-PEG and OM@MnO_2_-PEG in mouse serum (n = 3). **f** Release profiles of Mn^2+^ from OM@MnO_2_-PEG in buffer solution at different pH (n = 3). **g** The O_2_ concentration changes in acid solution (pH 6.5) with different concentrations of H_2_O_2_. Data are presented as Mean ± SEM

In this study, OMVs were collected and biomineralized by manganese oxide (MnO_2_), obtaining OM@MnO_2_-PEG nanoparticles for enhanced radio-immunotherapy of tumor via increasing the RT-caused ICD and cGAS-STING activation. In the acidic tumor microenvironment, OM@MnO_2_-PEG nanoparticles could react with hydrogen peroxide (H_2_O_2_) and then produced a plenty of Mn^2+^, O_2_ and OM fragments. The relieved tumor hypoxia improved the radio-sensitivity of tumor, amplifying RT-caused ICD and cGAS-STING activation. Meanwhile, oxygen/OMs promoted the macrophage polarization to anti-tumor M1 type and OM fragments enhanced the efficiency of tumor antigen presentation via the recognition of lipopolysaccharide (LPS) and toll-like receptors (TLRs). Notably, the prolonged high content of manganese ions in the tumor maintained the activated condition of cGAS-STING pathway. These comprehensive factors made OM@MnO_2_-PEG significantly enhance the immune stimulation effect of radiotherapy with high bio-safety. Especially, our yielding nanoparticles could improve the immune stimulation of both of EBRT and RNT, and the enhancement degree showed some difference. When the same local treatment outcome was obtained by EBRT and RNT, the distant anti-tumor effect of RNT was better than that of EBRT, which was related to the cGAS-STING pathway activation. In detail, cGAS-STING pathway activation was high and then gradually decreased after EBRT, but remained high in 7 days after ^131^I-mediated-RNT Fig. 1a. Therefore, this work developed a novel biomaterial for improving the immune stimulation effect of EBRT and RNT, and discovered some differences in cGAS-STING pathway activation between EBRT and RNT, providing some reference and strategic design for the implementation of clinical tumor RT.

## Results and discussion

### The preparation and characterization of OM@MnO_2_-PEG

OMVs were extracted from attenuated salmonella typhimurium VNP20009 through ultracentrifugation and then modified with 1, 2-distearoyl-sn-glycero-3-phosphoethanolamine-n-(polyethylene glycol)-5000 (DSPE-PEG_5000_), yielding OM-PEG nanoparticles. Then, with reference to the reported literatures, [47–49] manganese ions were loaded onto the OMs via a biomineralization process to obtain OM@MnO_2_-PEG. Additionally, this nanoparticle could be labeled by radio-iodine Fig. 1b. The collected OMVs possessed lipid-bilayered vesicular structures with the size of 20–200 nm observed by transmission electron microscope (TEM) **Figure S1a**. In contrast, OM@MnO_2_-PEG showed metallic inorganic matter on the broken OMVs Fig. 1c. The destruction of the complete vesicle structure might be attributed to the hypotonic and ultra-sonication treatments during the preparation of OM@MnO_2_-PEG. Furthermore, elemental mapping of OM@MnO_2_-PEG was carried out and the overlapping of manganese and oxygen indicated the successful formation of MnO_2_ on OMs Fig. 1d. Meanwhile, UV–Vis-NIR absorption spectrum of OM@MnO_2_-PEG showed the characteristic absorption peaks of OM-PEG and MnO_2_, doubly confirming the successful biomineralization of MnO_2_ onto OMs Figure S1b. The dynamic light scattering (DLS) analyses discovered that the hydrated particle size peak of OMVs and OM@MnO_2_-PEG was about 25 nm and 60 nm, respectively Figure S1c. The SDS-PAGE of OM-PEG and OM@MnO_2_-PEG showed the almost identical protein bands, illustrating the presence of OM protein in OM@MnO_2_-PEG Figure S2.

Finally, in order to test the radioactive iodine labeling stability of OM@MnO_2_-PEG, ^131^I-labeled this nanoparticle was incubated in serum and the deciduous ^131^I was further measured by a γ-counter. Most of the labeled iodine stayed stable on the nanoparticles after repeated cleaning and centrifugation, indicating our obtaining nanoparticle could be used as an effective carrier of radionuclide iodine Fig. 1e. Besides, the release of Mn^2+^ and production of O_2_ when OM@MnO_2_-PEG was incubated at different pH and H_2_O_2_ content were checked. It has been reported that MnO_2_ could react with H_2_O_2_ in the acidic condition, leading to the consumption of H_2_O_2_ and generation of Mn^2+^/O_2_ [47–49]. As shown in Fig. 1f, g, Mn^2+^ could be quickly released and significant amount of O_2_ was immediately produced at low pH/high H_2_O_2_ conditions, suggesting that OM@MnO_2_-PEG might be given off OM fragments, Mn^2+^ and O_2_ in tumor microenvironment.

### The radio-immunotherapy sensitization effect of OM@MnO_2_-PEG in vitro

**Fig. 2:**
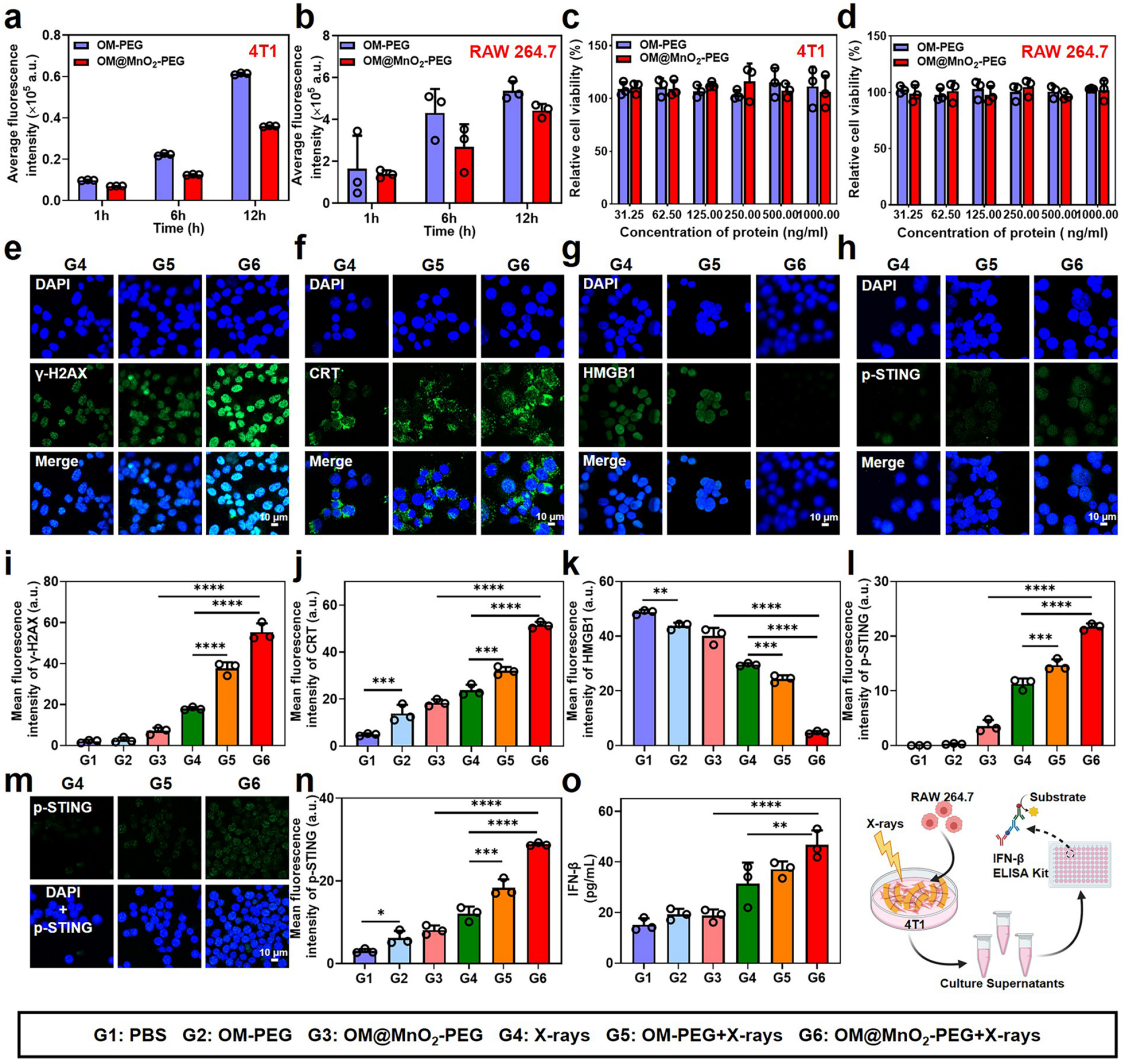
The radio-immunotherapy sensitization effect of OM@MnO_2_-PEG in vitro. **a**, **b** Flow cytometry analysis of 4T1 cells and RAW 264.7 cells incubated with DID-labeled OM-PEG or OM@MnO_2_-PEG (n = 3). **c**, **d** Relative cell viability of 4T1 cells and RAW 264.7 cells treated with different concentration of OM-PEG or OM@MnO_2_-PEG (n = 3). Fluorescent images of γ-H2AX (**e**), CRT (**f**), HMGB1 (**g**) and p-STING (**h**) expression in 4T1 cells with the indicated treatments (scale bar: 10 μm). Corresponding semi-quantitative analysis of γ-H2AX (**i**), CRT (**j**), HMGB1 (**k**) and p-STING (**l**) fluorescence intensity in 4T1 cells with the indicated treatments (n = 3). **m**, **n** Fluorescent images and corresponding statistical analysis of the expression of p-STING in RAW 264.7 cells with the indicated treatments (n = 3). **o** The content of IFN-β in the culture supernatant with indicated treatments measured by ELISA (n = 3). Schematic diagram showing the detection of IFN-β in the culture supernatants was given out. G1: PBS; G2: OM-PEG; G3: OM@MnO_2_-PEG; G4: X-rays; G5: OM-PEG + X-rays; G6: OM@MnO_2_-PEG + X-rays. Data are presented as Mean ± SD. Statistical significance was analyzed by one-way analysis of variance (ANOVA) with the least significant difference post hoc test (*P < 0.05, **P < 0.01, ***P < 0.001 and ****P < 0.0001)

Furthermore, the anti-tumor activity of OM@MnO_2_-PEG was tested in vitro. Firstly, the cell uptake of OM@MnO_2_-PEG by tumor cells and macrophage cells was measured. OM-PEG could be extensively taken in by both of 4T1 cells and RAW 264.7 cells in a time-dependent manner and the MnO_2_ modification slightly reduced the cell uptake of the nanoparticle, reflecting that OM@MnO_2_-PEG could be used as an effective drug delivery system Fig. 2a, b. The potential cytotoxicity of OM-PEG and OM@MnO_2_-PEG was studied by CCK-8 assay. Both of them showed no notable cytotoxicity to 4T1 cells and RAW 264.7 cells at our test concentrations, indicating the great biocompatibility of OM@MnO_2_-PEG Fig. 2c, d. Considering the oxygen generation from the reaction between OM@MnO_2_-PEG and H_2_O_2_, OM@MnO_2_-PEG nanoparticle was used to enhance the radio-sensitivity of 4T1 cells. The immunofluorescence intensity of γ-H2AX foci, a biomarker of DNA double-strand breaks, was used to study the extent of DNA damage induced by X-ray treatment. Notably, the immunofluorescence intensity in the 4T1 cells treated by OM@MnO_2_-PEG plus RT was strongest, proving that OM@MnO_2_-PEG was efficient radio-sensitizer Fig. 2e and i, Figure S3a. Moreover, owing to the existence of OM and oxygen, RT-induced ICD might be improved. The expression of calreticulin (CRT) and the release of high mobility group protein B1 (HMGB1) were recognized as the markers of ICD from dying cells. As shown by confocal microscopic images, 4T1 cells treated with OM@MnO_2_-PEG and RT showed significant expression of CRT on the surface of 4T1 cells and obvious release of HMGB1 from the nucleus Fig. 2f, g, j and k, Figure S3b&c. In this way, OM@MnO_2_-PEG enhanced the anti-tumor effect of RT as well as RT-induced ICD.

Additionally, RT can cause the release of damaged DNA fragments from the nucleus/mitochondria into the cytoplasm, further activating cGAS-STING pathway [11, 12]. Mn^2+^, which can be released from OM@MnO_2_-PEG in tumor acidic microenvironment, is also proved to enhance cGAS-STING activation [44, 47, 48]. Therefore, we next investigated whether OM@MnO_2_-PEG could enlarge cGAS-STING pathway activation under X-rays exposure in both of 4T1 cells and RAW 264.7 cells. Immunofluorescence was used for validation of the expression of p-STING, which is an activated protein in the cGAS-STING dependent signaling pathway. OM@MnO_2_-PEG plus RT promoted the obvious up-regulation of p-STING expression in both of 4T1 cells and RAW 264.7 cells, demonstrating that OM@MnO_2_-PEG plus RT could indeed activate cGAS-STING pathway Fig. 2h, l, m and n, Figure S3d, S4. Moreover, the content of interferon-β (IFN-β) as a result of cGAS-STING pathway activation was tested by enzyme linked immune-sorbent assay (ELISA). The results showed that OM@MnO_2_-PEG plus RT could increase the secretion of IFN-β Fig. 2o. Taken together, OM@MnO_2_-PEG could not only enhance the radio-sensitivity of tumor cells, but also strengthen RT-induced ICD and cGAS-STING pathway activation.

### The tumor microenvironment regulation by OM@MnO_2_-PEG

**Fig. 3:**
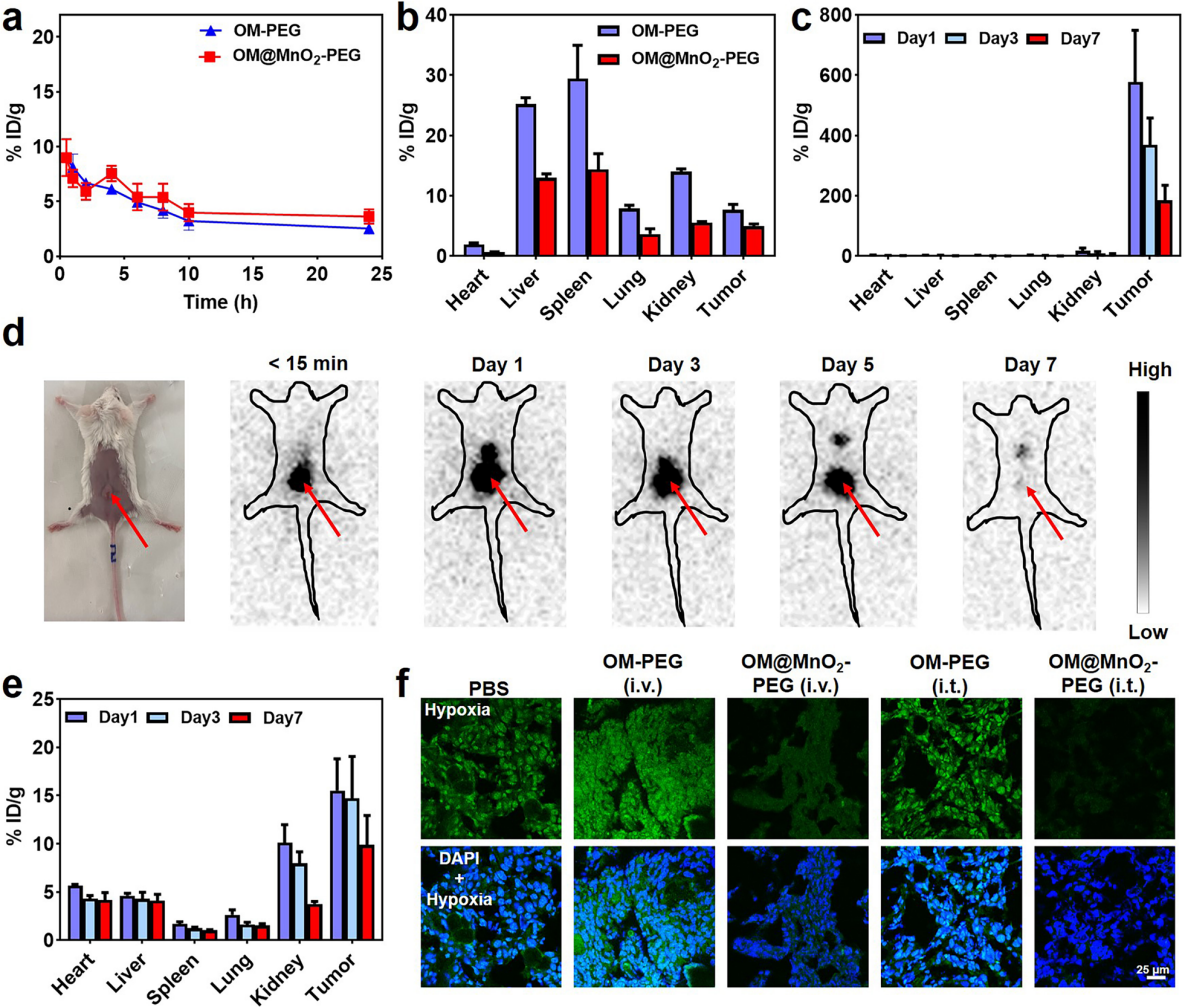
The in vivo behavior and tumor microenvironment regulation of OM@MnO_2_-PEG**. a** The blood circulation profiles of ^131^I-OM-PEG and ^131^I-OM@MnO_2_-PEG (n = 3). **b** The bio-distribution of ^131^I-OM-PEG and ^131^I-OM@MnO_2_-PEG measured at 24 h post intravenous injection (n = 3). **c** The bio-distribution of ^131^I-OM@MnO_2_-PEG measured at 1, 3 and 7 d post intratumor injection (n = 3). **d** The SPECT images of the mouse at 0, 1, 3, 5 and 7 d post intratumor injection of ^131^I-OM@MnO_2_-PEG. **e** Distribution of manganese at 1, 3 and 7 d post intratumor injection of OM@MnO_2_-PEG (n = 3). **f** fluorescent images of hypoxia in 4T1 tumors with the indicated treatments (scale bar: 25 μm). Data are presented as mean ± SEM

Motivated by the perfect sensitizing effect on radio-immunotherapy of OM@MnO_2_-PEG in vitro, we performed the enhanced radio-immunotherapy of tumor in vivo using this nanoparticle. Firstly, in order to understand the in vivo behaviors of OM@MnO_2_-PEG, we quantitatively studied the blood circulation and bio-distribution profiles of ^131^I-OM@MnO_2_-PEG. ^131^I-OM@MnO_2_-PEG was intravenously injected into 4T1 tumors-bearing mice and the blood was collected from these mice at appointed time (0.5, 1, 2, 4, 6, 8, 10 and 24 h) post injection. Then, the radioactivity of these blood samples was determined by a γ-counter. As shown in Fig. 3a, the blood circulation half-life of ^131^I-OM@MnO_2_-PEG was long and almost similar with that of ^131^I-OM-PEG. For the bio-distribution study, the major organs, including heart, liver, spleen, lung and kidney, and tumors were collected at 24 h post intravenous injection, and their weight and radioactivity were recorded. The tumor accumulation of OM-PEG and OM@MnO_2_-PEG was 7.65% ± 1.60% and 4.94% ± 0.66%, and their liver accumulation was 25.22% ± 1.83% and 12.99% ± 1.12%, suggesting the good tumor-targeting capacity of OM-PEG and OM@MnO_2_-PEG Fig. 3b. However, the amount of Mn^2+^ delivered by OM@MnO_2_-PEG to the tumor was limited and would gradually decrease over time after intravenous injection. This tumor-targeting behavior could image the tumors and outline their location to guide the follow-up local drug administration and RT implement. Optionally, OM@MnO_2_-PEG could be injected intratumorally with the help of advanced imaging techniques and interventional methods. ^131^I-OM@MnO_2_-PEG exhibited good tumor-specific retention and low off-target toxicity in one week post intratumoral injection Fig. 3c. Consistent to the result of bio-distribution study, SPECT images showed that ^131^I-OM@MnO_2_-PEG had a strong tumor retention ability after intratumoral injection Fig. 3d. Sequentially, inductively coupled plasma-mass spectrometry (ICP-MS) was used to quantitatively examine the bio-distribution of OM@MnO_2_-PEG after being administrated intratumorally by detecting the Mn content in tumors and major organs. A plenty of Mn^2+^ was accumulated in the tumor in one week, which was benefit to maintain the activation of cGAS-STING pathway. In addition, a part of Mn^2+^ was existed in the kidney, indicating the degradation of OM@MnO_2_-PEG in the acidic tumor microenvironment Fig. 3e. With the reaction between OM@MnO_2_-PEG and H_2_O_2_, oxygen could be produced in the tumor. Therefore, we checked the tumor hypoxia after different treatments. OM@MnO_2_-PEG could relieve the hypoxic microenvironment of the tumor and the effect of intratumoral injection was more effective than that of intravenous injection. Fig. 3f**, Figure S5**.

### The tumor growth inhibition by OM@MnO_2_-PEG-mediated RT

**Fig. 4:**
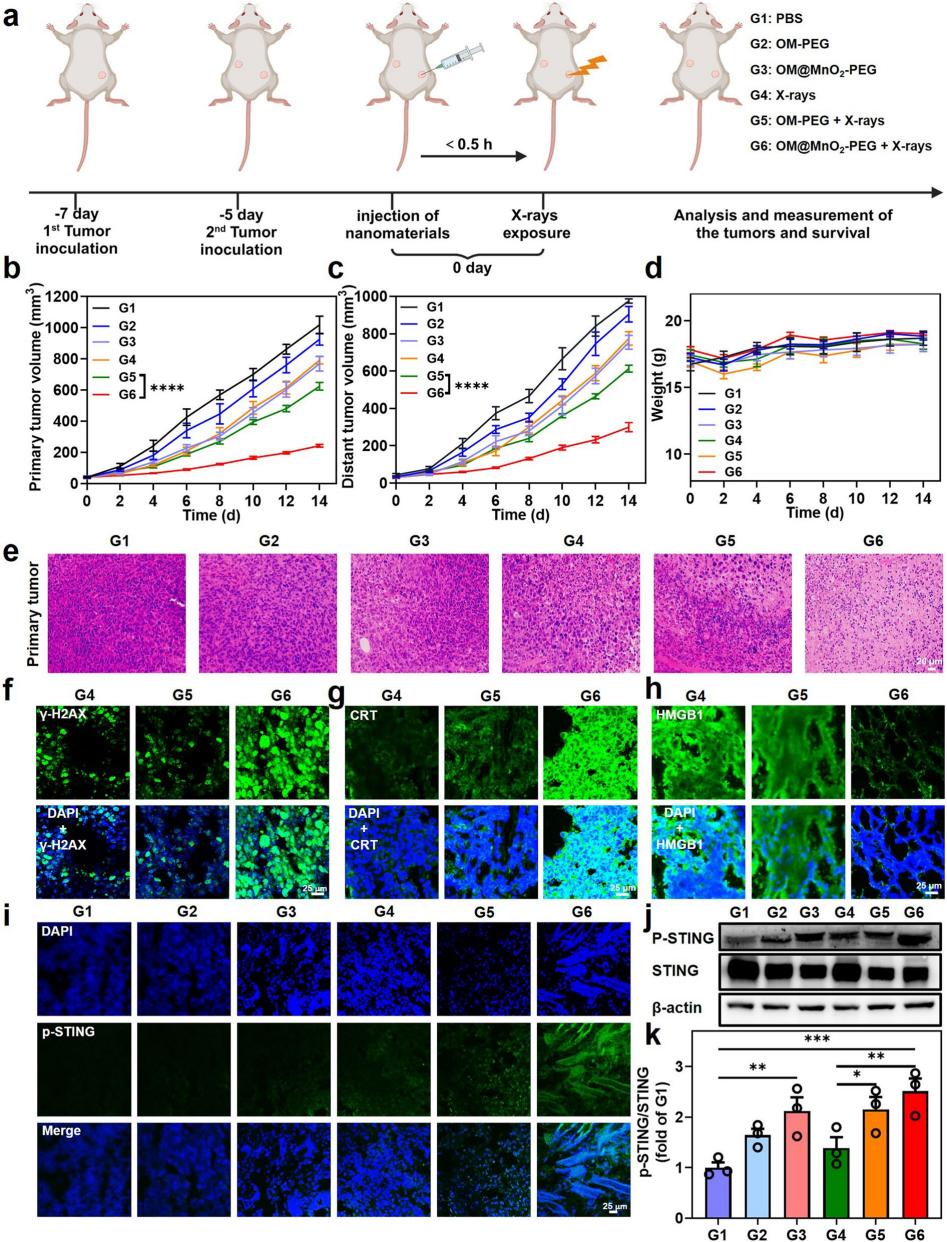
The tumor growth inhibition by OM@MnO_2_-PEG-mediated RT. **a** Schematic diagram of the experimental schedule. The dose of X-rays was 6 Gy. **b**, **c** Primary and distant tumor growth curves of mice with indicated treatments (n = 5). **d** Average body weights of 4T1 tumors-bearing mice after various treatments (n = 5). **e** H&E staining of primary tumor sections with various treatments (scale bar: 20 μm). Fluorescent images of γ-H2AX (**f**), CRT (**g**), HMGB1 (**h**) and p-STING (**i**) expression in primary tumor sections with the indicated treatments (scale bar: 25 μm). **j**, **k** Representative western blotting images and corresponding statistical analysis of the expression of p-STING/STING in primary tumor with the indicated treatments (n = 3). Data are presented as Mean ± SEM. Statistical significance was analyzed by one-way analysis of variance (ANOVA) with the least significant difference post hoc test. (*P < 0.05, **P < 0.01, ***P < 0.001 and ****P < 0.0001)

Next, we explored the tumor radio-sensitization ability of OM@MnO_2_-PEG and the anti-tumor immune-stimulatory effect of OM@MnO_2_-PEG-mediated RT in vivo. Thirty mice bearing subcutaneous bilateral 4T1 tumors were divided into the following six groups at random (n = 5 per group): G1: PBS; G2: OM-PEG; G3: OM@MnO_2_-PEG; G4: X-rays; G5: OM-PEG + X-rays; G6: OM@MnO_2_-PEG + X-rays. The dose of X-rays was 6 Gy and the dose of OMs was 5 μg protein per mouse. The treatment schedule was showed in Fig. 4a. The primary tumors were exposed to the X-rays in half an hour post intratumoral injection of the nanoparticles. Then the tumor size was measured with a vernier caliper and the body weight of mouse was measured every two days. According to the tumor growth curves of primary tumors, OM@MnO_2_-PEG had a certain inhibitory effect on the primary tumor and it could enhance the therapeutic effect of X-rays exposure, demonstrating OM@MnO_2_-PEG was an effective radio-sensitizer Fig. 4b. Moreover, this enhanced RT inhibited the distant tumor growth to some extent, owing to the RT-induced anti-tumor immune-stimulatory effect Fig. 4c. Additionally, there was no remarkable loss in the body weight of mice, suggesting the bio-safety of our therapeutic strategy Fig. 4d. Meanwhile, the hematoxylin–eosin (H&E) stained images showed no obvious toxicity of our treatment strategy to the normal tissues, including heart, liver, spleen, lung and kidney Figure S6. The level of cell damage and apoptosis of the primary/distant tumor sections were also analyzed by H&E staining. As predicted, the treatment of OM@MnO_2_-PEG and X-rays could induce the most serious damage to the tumor cells in both of primary and distant tumor tissue, confirming the excellent radio-sensitization effect of OM@MnO_2_-PEG Fig. 4e, Figure S7.

RT induced the anti-tumor effect by directly killing the tumor cells and triggering the systemic immune response. Meanwhile, biomaterials could be designed to strengthen both of the cell-killing capacity and the positive anti-tumor immune stimulation of RT. Firstly, RT could cause DNA damage and ICD of primary tumors and OM@MnO_2_-PEG could amplify these effects. We performed the immunofluorescence staining (γ-H2AX, CRT and HMGB1) assay of the primary tumors. OM@MnO_2_-PEG injection or X-rays exposure could enhance the expression of γ-H2AX/CRT and promote the migration of HMGB1, and OM@MnO_2_-PEG-mediated RT showed the strongest ability to cause these changes Fig. 4f, g and h, Figure S8. Moreover, p-STING expression in the primary tumors was analyzed by immunofluorescence and western blotting. The results showed that OM@MnO_2_-PEG could promote the RT-induced cGAS-STING activation, which could further strengthen RT-triggered anti-tumor systemic immunotherapy of tumor Fig. 4i, j and k, Figure S9. Different with other biomaterials, OM@MnO_2_-PEG combined the respective advantage of OM and MnO_2_ in radio-immunotherapy sensitization. This nanoparticle could produce/release OM, Mn^2+^ and O_2_ in the tumor microenvironment. O_2_ improved the treatment outcome of both of radiotherapy and immunotherapy [47–49]. Mn^2+^ can synergy with O_2_-enhanced radiotherapy to activate the cGAS-STING pathway [49]. Moreover, OM plays a key role in many aspects of innate and adaptive immunity [40]. Therefore, OM@MnO_2_-PEG could enhance the direct toxicity and the efficacy of immune activation of RT.

### Phenotype analysis of immune cells

**Fig. 5:**
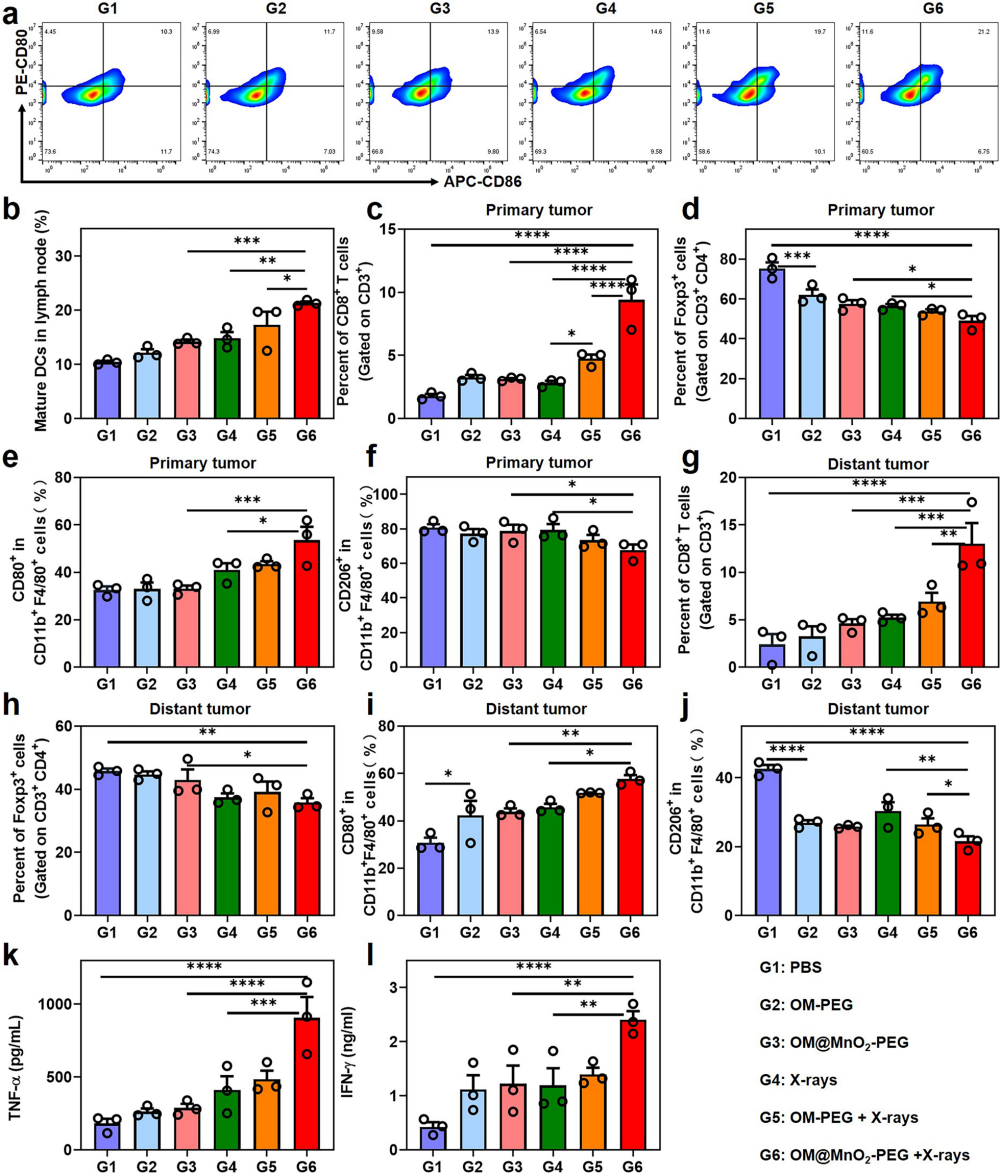
Phenotype analysis of immune cells. **a**, **b** Representative flow cytometry plots and quantification of DC maturation in lymph nodes of 4T1 tumor-bearing mice with various treatments (gated on CD11c^+^ DC cells) at day 3 post injection (n = 3). **c** Percentage of tumor-infiltrating CD8^+^ T cells among CD3^+^ cells in primary tumors with different treatments at day 3 post injection (n = 3). **d** Proportion of Treg cells gated on CD3^+^CD4^+^ T cells in primary tumors at day 3 post injection (n = 3). **e**, **f** Proportion of M1 macrophages (CD11b^+^F4/80^+^CD80^+^) and M2 macrophages (CD11b^+^F4/80^+^CD206^+^) in primary tumors at day 3 post injection (n = 3). **g** Percentage of tumor-infiltrating CD8^+^ T cells among CD3^+^ cells in distant tumors with different treatments at day 3 post injection (n = 3). **h** Proportion of Treg cells gated on CD3^+^CD4^+^ T cells in distant tumors at day 3 post injection (n = 3). **i**, **j** Proportion of M1 macrophages (CD11b^+^F4/80^+^CD80^+^) and M2 macrophages (CD11b^+^F4/80^+^CD206^+^) in distant tumors at day 3 post injection (n = 3). **k**, **l** The concentration of TNF-α and IFN-γ in serum were analyzed at day 3 after indicated treatments (n = 3). Data are presented as Mean ± SEM. Statistical significance was analyzed by one-way analysis of variance (ANOVA) with the least significant difference post hoc test (*P < 0.05, **P < 0.01, ***P < 0.001 and ****P < 0.0001)

The enhanced ICD and cGAS-STING signaling pathway in the process of RT could promote the formation of an “in-situ” tumor vaccine and reversal of the inhibitory immune microenvironment, further changing the phenotype of various immune cells. To clarify the mechanism, 6 groups of Balb/c mice bearing subcutaneous bilateral 4T1 tumors were prepared and sacrificed 3 days after indicated treatments. The bilateral tumors and adjacent groin inguinal lymph nodes close to the primary tumors were collected for phenotype analysis of immune cells by flow cytometer. The immunoassay of DC maturation was firstly performed. Compared with other treatments, OM@MnO_2_-PEG-mediated RT significantly enhanced the frequency of DC maturation, demonstrating the satisfactory presentation of the tumor antigen in our anti-tumor strategy Fig. 5a, b Figure S10. Next, we checked the proportion of adaptive immune cells, mainly including CTLs and regulatory T cells (Tregs), and innate immunocytes, including M1-type macrophages and M2-type macrophages, in both of primary and distant tumors. The treatment of OM@MnO_2_-PEG plus X-rays increased the infiltration of CD8^+^ T cells into primary tumor, while the proportion of immunosuppressive Tregs (CD3^+^ CD4^+^ Foxp3^+^) was decreased in CD4^+^ T cells after this treatment Fig. 5c, d, Figure S11, S12. Additionally, the similar tendency of immune analysis about the CD8^+^ T cells and Tregs were demonstrated in the distant tumors, suggesting that our treatment strategy could induce powerful and systemic T cells-involved anti-tumor immunity Fig. 5g, h, Figure S13, S14. Moreover, OM@MnO_2_-PEG-mediated RT could also activate the innate anti-tumor immunity by promoting the macrophage polarization to M1 in the primary and distant tumors Fig. 5e, f, i and j, Figure S15, S16. This kind of macrophage polarization might be attributed to the integrated function of O_2_, LPS of OMs and cGAS-STING activation in the tumor microenvironment. Taken together, the strong T cells and macrophage-mediated anti-tumor immune response could be realized in mice treated with OM@MnO_2_-PEG-mediated RT. Besides, the content of anti-tumor cytokines, including tumor necrosis factor-α (TNF-α) and interferon-γ (IFN-γ), was also increased after OM@MnO_2_-PEG-mediated RT, indicating the existence of systemic anti-tumor immunity in mice treated with our strategy Fig. 5k, l. In our study, OM@MnO_2_-PEG released OM fragments, Mn^2+^ and O_2_ in the tumor microenvironment. The relieved tumor hypoxia made tumor cells sensitive to RT, resulting in severe ICD and DNA damage of tumor. DNA fragments in cytoplasm together with detained Mn^2+^ caused the activation of cGAS-STING, which played an important role in DC maturation, the proliferation/tumor invasion of CTLs/Tregs and macrophage polarization. Meanwhile, the production of O_2_ and OM fragments in the tumor tissue could also make contribution to enhance the proportion of anti-tumor M1. In this way, OM@MnO_2_-PEG-mediated RT realized multi-stage and multi-angle activation of adaptive immunity and innate immunity.

### Comparison of the tumor growth inhibition between OM@MnO_2_-PEG-mediated EBRT and RNT

**Fig. 6:**
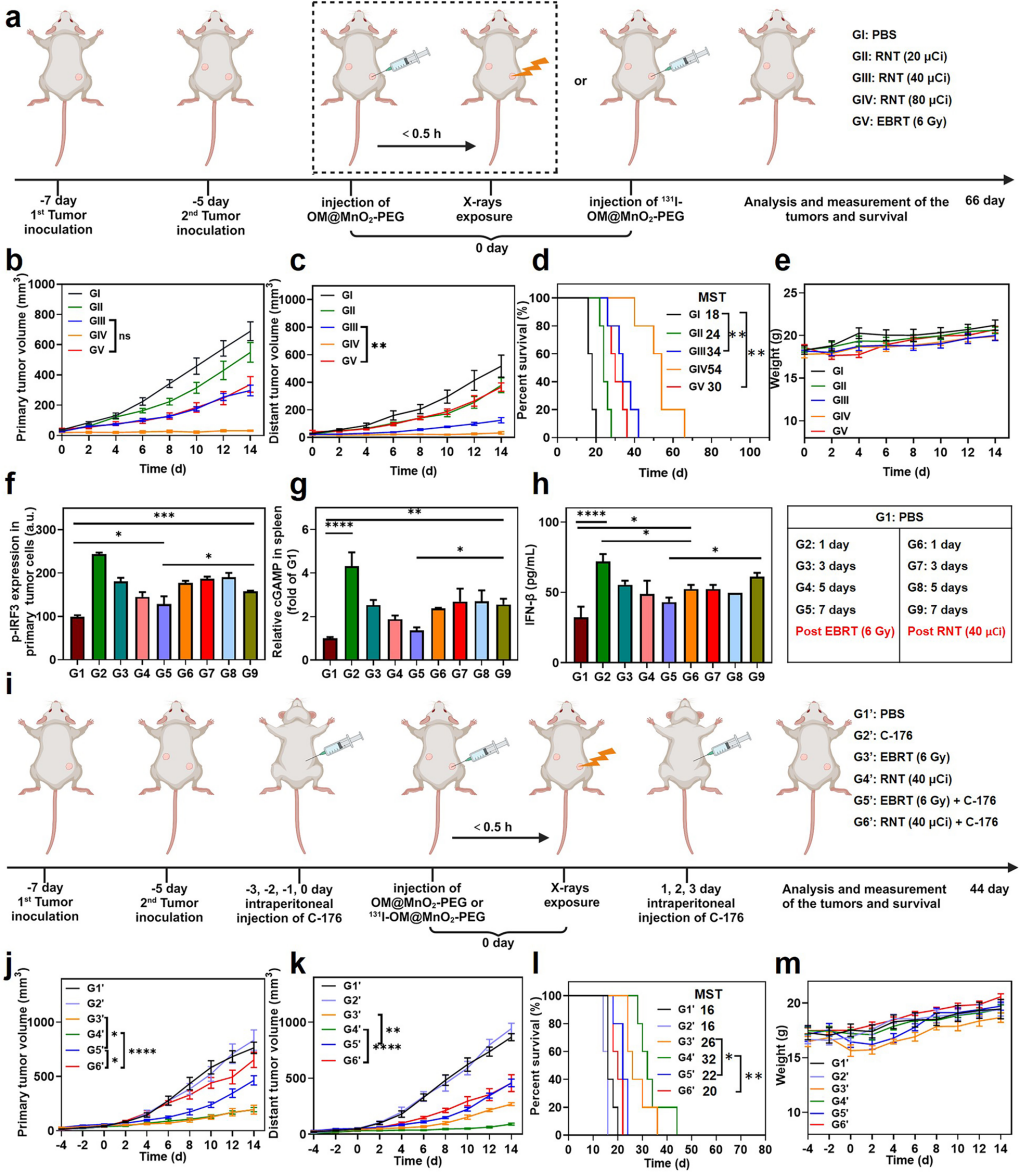
Comparison of the tumor growth inhibition between OM@MnO_2_-PEG-mediated EBRT and RNT. **a** Schematic diagram of the experimental schedule of EBRT and RNT. **b**, **c** Primary and distant tumor growth curves of 4T1 tumor-bearing mice after various treatments (n = 5). **d** The survivorship curves of 4T1 tumor-bearing mice after various treatments (n = 5). **e** Average body weights of 4T1 tumors-bearing mice after various treatments (n = 5). MST indicated median survival time. **f** The expression of p-IRF in primary tumor tissues with indicated treatments measured by flow cytometer (n = 3). **g** The expression of cGAMP in spleens with indicated treatments measured by ELISA (n = 3). **h** The expression of IFN-β in serum with indicated treatments measured by ELISA (n = 3). **i** Schematic diagram of the experimental schedule of C-176 inhibition. **j**, **k** Primary and distant tumor growth curves of 4T1 tumor-bearing mice after various treatments (n = 5). **l** The survivorship curves of 4T1 tumor-bearing mice after various treatments (n = 5). MST indicated median survival time. **m** Average body weights of 4T1 tumors-bearing mice after various treatments (n = 5). Data are presented as Mean ± SEM. Statistical significance was analyzed by one-way analysis of variance (ANOVA) with the least significant difference post hoc test. Statistical significance of survivorship curves was analyzed by log-rank test (*P < 0.05, **P < 0.01, ***P < 0.001 and ****P < 0.0001)

RT is divided into EBRT and RNT, according to the different radiation sources. EBRT and RNT shows similar biological mode of action and pathway of sensitization. The irradiation exposure in EBRT is transient and intense, but irradiation exposure in RNT is constant and gentle. Whether the immune stimulation of these two kind of RT has some different characteristics is not clear. Therefore, we preformed ^131^I-OM@MnO_2_-PEG-mediated RNT and compared the anti-tumor effects between OM@MnO_2_-PEG-mediated EBRT and RNT. We labelled ^131^I onto OM@MnO_2_-PEG nanoparticles at different radioactive dose. Mice were divided into five groups: GI: PBS; GII: RNT (20 μCi); GIII: RNT (40 μCi); GIV: RNT (80 μCi); GV: EBRT (6 Gy). The mice were treated as shown in the Fig. 6a, and ^131^I-OM@MnO_2_-PEG or OM@MnO_2_-PEG was injected intratumorally. With the increase of the dose of radionuclide, the therapeutic effect of RNT on primary and distant tumors was gradually enhanced, showing the powerful tumor-killing and anti-tumor immunity-stimulating capacity of RNT. Moreover, RNT (40 μCi) exhibited equivalent inhibitory effect on the primary tumors to EBRT (6 Gy), but showed better inhibitory effect than EBRT (6 Gy) on the distant tumors, suggesting that RNT had better immune stimulation effect than EBRT Fig. 6b, c. In addition, the treatment of RNT and EBRT prolonged the survival period of tumor-bearing mice Fig. 6d. The body weight of mice was not remarkably decreased after various treatments, suggesting the perfect bio-safety of these therapeutic strategies Fig. 6e. EBRT took potent effect in a short period of time, while RNT gradually took effect. The time course of RT-induced DNA damage might affect the degree and duration of cGAS-STING activation, further regulating the anti-tumor immunity. Therefore, we compared the degree of the cGAS-STING activation in one week after treatments EBRT and RNT. In detail, we investigated the expression of p-IRF-3 in tumors, cGAMP in spleens and IFN-β in the serum at day 1, 3, 5 and 7 after EBRT or RNT measured by flow cytometer or ELISA. The p-IRF3/cGAMP was highly expressed at day 1, and then got gradually low at day 3, 5 and 7 in the mice treated with EBRT. In contrast, the expression of p-IRF3/cGAMP kept relatively high and stable in one week in the mice treated with RNT Fig. 6f, g. Similarly, the content of IFN-β in the serum presented the same tendency in mice treated with EBRT or RNT Fig. 6h. Taken together, both of RNT and EBRT could enhance the activation of cGAS-STING and RNT showed a relative longer-term effect on cGAS-STING pathway activation than that of EBRT.

To further validate the participation of cGAS-STING activation in the therapeutic effect of EBRT and RNT, STING inhibitor (C-176) was selected to block the function of cGAS-STING pathway Fig. 6i. Mice were divide into six groups: G1′: PBS; G2′: C-176; G3′: EBRT (6 Gy); G4′: RNT (40 μCi); G5′: EBRT (6 Gy) + C-176; G6′: RNT (40 μCi) + C-176. C-176 significantly promoted the tumor growth of primary and distant tumors in mice treated with EBRT or RNT, indicating the important role of cGAS-STING activation in anti-tumor therapeutic effect of RT Fig. 6j, k. More importantly, the growth of primary tumors in mice treated with C-176 plus RNT was faster than that in mice treated with C-176 plus EBRT, suggesting the therapeutic effect of RNT was more dependent on cGAS-STING activation than that of EBRT. The survivorship curves also showed that C-176 injection decreased the anti-tumor ability of EBRT and RNT Fig. 6l. Finally, the change of average body weight in each group confirmed the safety of OM@MnO_2_-PEG-mediated ENRT and RNT Fig. 6m. The cGAS-STING activation-involved tumor immune microenvironment was closely related to the efficacy of immunotherapy. Besides, the difference in anti-tumor immune stimulation triggered by EBRT and RNT might be also affected by other factors, such as ICD enhancement, toxicity of radiation to immune cells and the radiation-induced other changes in tumor microenvironment [3, 4, 8, 9, 16]. Notably, the effect of various biomaterials on the sensitizing ability of radio-immunotherapy during EBRT and RNT may be different. In this study, the continuous production of oxygen from OM@MnO_2_-PEG could enhance the induction of ICD and DNA damage over a long time during RNT, while over a short time during EBRT. Meanwhile, the direct killing of tumor infiltrating immune cells was persistent during RNT and temporary during EBRT. These differences might further cause the different therapeutic effect of radio-immunotherapy, which need more studies to elucidate.

## Conclusion

In summary, we successfully extracted OMVs through ultracentrifugation and loaded MnO_2_ onto OMs through biomineralization, yielding OM@MnO_2_-PEG nanoparticles. In the tumor microenvironment with low pH and high H_2_O_2_ content, this nanoparticle could release OM fragments, O_2_ and Mn^2+^. The generated O_2_ relieved the hypoxic tumor microenvironment and alleviated the radiation resistance of tumor tissue. Under X-rays exposure, ICD of the tumor cells would be caused, and a plenty of DNA was damaged and leaked into the cytoplasm, further activating the cGAS-STING pathway. This RT-induced ICD could be amplified by O_2,_ and OM fragments enhanced the antigen presenting ability of DCs. More importantly, the released Mn^2+^ could directly bind to cGAS to enhance the binding affinity between cGAS and cytoplasmic DNA, thereby making cells more sensitive to activate the downstream STING pathway and finally producing strong anti-tumor immunity even in the presence of trace amounts of cytoplasmic DNA. Besides, all of O_2_, OMs and cGAS-STING activation could promote the polarization of macrophages towards anti-tumor M1. Notably, compared to EBRT, RNT could generate more persistent and powerful cGAS-STING activation, and then triggered much stronger systemic anti-tumor immunity. Therefore, a novel nanoparticle was obtained in this study to enhance the direct killing ability and the immune stimulation effect of RT, and this nanoparticle-mediated EBRT and RNT showed differential reinforcement of cGAS-STING pathway-involved immunotherapy.

### Supplementary Information

Additional file 1.

## Data Availability

Data will be made available on request.

## References

[1] Jagodinsky JC, Jin WJ, Bates AM, Hernandez R, Grudzinski JJ, Marsh IR, Chakravarty I, Arthur IS, Zangl LM, Brown RJ, Nystuen EJ, Emma SE, Kerr C, Carlson PM, Sriramaneni RN, Engle JW, Aluicio-Sarduy E, Barnhart TE, Le T, Kim K, Bednarz BP, Weichert JP, Patel RB, Morris ZS (2021). Temporal analysis of type 1 interferon activation in tumor cells following external beam radiotherapy or targeted radionuclide therapy. Theranostics.

[2] Grzmil M, Boersema P, Sharma A, Blanc A, Imobersteg S, Pruschy M, Picotti P, Schibli R, Behe M (2022). Comparative analysis of cancer cell responses to targeted radionuclide therapy (TRT) and external beam radiotherapy (EBRT). J Hematol Oncol.

[3] Pan S, Huang G, Sun Z, Chen X, Xiang X, Jiang W, Xu Y, Chen T, Zhu X (2023). X-ray-responsive zeolitic imidazolate framework-capped nanotherapeutics for cervical cancer-targeting radiosensitization. Adv Funct Mater.

[4] Zhang Z, Liu X, Chen D, Yu J (2022). Radiotherapy combined with immunotherapy: the dawn of cancer treatment. Signal Transduct Tar.

[5] Gao S, Li T, Guo Y, Sun C, Xianyu B, Xu H (2020). Selenium-containing nanoparticles combine the NK cells mediated immunotherapy with radiotherapy and chemotherapy. Adv Mater.

[6] McLaughlin M, Patin EC, Pedersen M, Wilkins A, Dillon MT, Melcher AA, Harrington KJ (2020). Inflammatory microenvironment remodelling by tumour cells after radiotherapy. Nat Rev Cancer.

[7] Cytlak UM, Dyer DP, Honeychurch J, Williams KJ, Travis MA, Illidge TM (2022). Immunomodulation by radiotherapy in tumour control and normal tissue toxicity. Nat Rev Immunol.

[8] Sun L, Shen F, Tian L, Tao H, Xiong Z, Xu J, Liu Z (2021). ATP-responsive smart hydrogel releasing immune adjuvant synchronized with repeated chemotherapy or radiotherapy to boost antitumor immunity. Adv Mater.

[9] Deng Z, Liu J, Xi M, Wang C, Fang H, Wu X, Zhang C, Sun G, Zhang Y, Shen L, Chen M, Ji J, Liu Z, Yang G (2022). Biogenic platinum nanoparticles on bacterial fragments for enhanced radiotherapy to boost antitumor immunity. Nano Today.

[10] Duo Y, Chen Z, Li K, Yang Y, Wang H, Hu J, Luo G (2023). Targeted delivery of novel Au(I)-based AIEgen via inactivated cancer cells for trimodal chemo-radio-immunotherapy and vaccination against advanced tumor. Nano Today.

[11] Du S, Chen G, Yuan B, Hu Y, Yang P, Chen Y, Zhao Q, Zhou J, Fan J, Zeng Z (2021). DNA sensing and associated type 1 interferon signaling contributes to progression of radiation-induced liver injury. Cell Mol Immunol.

[12] Liu N, Zhu J, Zhu W, Chen L, Li M, Shen J, Chen M, Wu Y, Pan F, Deng Z, Liu Y, Yang G, Liu Z, Chen Q, Yang Y (2023). X-ray-induced release of nitric oxide from hafnium-based nanoradiosensitizers for enhanced radio-immunotherapy. Adv Mater.

[13] Gu J, Liu X, Ji Z, Shen M, Zhu M, Ren Y, Guo L, Yang K, Liu T, Yi X (2023). Tumor vascular destruction and cGAS-STING activation induced by single drug-loaded nano-micelles for multiple synergistic therapies of cancer. Small.

[14] Xu W, Qiao L, Wang Z, Qian Y, Li L, Sun Q, Li C (2023). Dual-pathway STING activation and chemodynamic therapy for improved anti-tumor therapy. Chem Eng J.

[15] Cao L, Tian H, Fang M, Xu Z, Tang D, Chen J, Yin J, Xiao H, Shang K, Han H, Li X (2022). Activating cGAS-STING pathway with ROS-responsive nanoparticles delivering a hybrid prodrug for enhanced chemo-immunotherapy. Biomaterials.

[16] Pei P, Zhang Y, Jiang Y, Shen W, Chen H, Yang S, Zhang Y, Yi X, Yang K (2022). Pleiotropic immunomodulatory functions of radioactive inactivated bacterial vectors for enhanced cancer radio-immunotherapy. ACS Nano.

[17] Sang W, Zhang Z, Wang G, Xie L, Li J, Li W, Tian H, Dai Y (2022). A triple-kill strategy for tumor eradication reinforced by metal-phenolic network nanopumps. Adv Funct Mater.

[18] Wang C, Dong Z, Hao Y, Zhu Y, Ni J, Li Q, Liu B, Han Y, Yang Z, Wan J, Yang K, Liu Z, Feng L (2022). Coordination polymer-coated CaCO_3_ reinforces radiotherapy by reprogramming the immunosuppressive metabolic microenvironment. Adv Mater.

[19] Zhang P, Rashidi A, Zhao J, Silvers C, Wang H, Castro B, Ellingwood A, Han Y, Lopez-Rosas A, Zannikou M, Dmello C, Levine R, Xiao T, Cordero A, Sonabend AM, Balyasnikova IV, Lee-Chang C, Miska J, Lesniak MS (2023). STING agonist-loaded, CD47/PD-L1-targeting nanoparticles potentiate antitumor immunity and radiotherapy for glioblastoma. Nat Commun.

[20] Li S, Wang Y, Wang X, Feng J, Guo DS, Meng Z, Liu Y, Sun SK, Zhang Z (2023). Macrocyclic-albumin conjugates for precise delivery of radionuclides and anticancer drugs to tumors. ACS Nano.

[21] Pei P, Shen W, Zhou H, Sun Y, Zhong J, Liu T, Yang K (2021). Radionuclide labeled gold nanoclusters boost effective anti-tumor immunity for augmented radio-immunotherapy of cancer. Nano Today.

[22] Zai W, Kang L, Dong T, Wang H, Yin L, Gan S, Lai W, Ding Y, Hu Y, Wu J, E.  (2021). Coli membrane vesicles as a catalase carrier for long-term tumor hypoxia relief to enhance radiotherapy. ACS Nano.

[23] Wang H, Liu J, Zhu X, Yang B, He Z, Yao X (2023). AZGP1P2/UBA1/RBM15 cascade mediates the fate determinations of prostate cancer stem cells and promotes therapeutic effect of docetaxel in castration-resistant prostate cancer via TPM1 m6A modification. Research.

[24] Wang Q, Yang T, Li S, Xu C, Wang C, Xiong Y, Wang X, Wan J, Yang X, Li Z (2023). Unimolecular self-assembled hemicyanine–oleic acid conjugate acts as a novel succinate dehydrogenase inhibitor to amplify photodynamic therapy and eliminate cancer stem cells. Research.

[25] Zhang C, Yuan Y, Wu K, Wang Y, Zhu S, Shi J, Wang L, Li Q, Zuo X, Fan C, Chang C, Li J (2022). Driving DNA origami assembly with a terahertz wave. Nano Lett.

[26] Wang Y, Chen J, Duan R, Gu R, Wang W, Wu J, Lian H, Hu Y, Yuan A (2022). High-Z-sensitized radiotherapy synergizes with the intervention of the pentose phosphate pathway for in situ tumor vaccination. Adv Mater.

[27] Dai X, Ruan J, Guo Y, Sun Z, Liu J, Bao X, Zhang H, Li Q, Ye C, Wang X, Zhao C-X, Zhou F, Sheng J, Chen D, Zhao P (2021). Enhanced radiotherapy efficacy and induced anti-tumor immunity in HCC by improving hypoxia microenvironment using oxygen microcapsules. Chem Eng J.

[28] Dong Z, Wang C, Gong Y, Zhang Y, Fan Q, Hao Y, Li Q, Wu Y, Zhong X, Yang K, Feng L, Liu Z (2022). Chemical modulation of glucose metabolism with a fluorinated CaCO_3_ nanoregulator can potentiate radiotherapy by programming antitumor immunity. ACS Nano.

[29] Zhang C, Xia D, Liu J, Huo D, Jiang X, Hu Y (2020). Bypassing the Immunosuppression of myeloid-derived suppressor cells by reversing tumor hypoxia using a platelet-inspired platform. Adv Funct Mater.

[30] Huang H, Zhang C, Wang X, Shao J, Chen C, Li H, Ju C, He J, Gu H, Xia D (2020). Overcoming hypoxia-restrained radiotherapy using an erythrocyte-inspired and glucose-activatable platform. Nano Lett.

[31] Zhang C, Ren J, Hua J, Xia L, He J, Huo D, Hu Y (2018). Multifunctional Bi_2_WO_6_ nanoparticles for CT-guided photothermal and oxygen-free photodynamic therapy. ACS Appl Mater Interfaces.

[32] Zuo H, Tao J, Shi H, He J, Zhou Z, Zhang C (2018). Platelet-mimicking nanoparticles co-loaded with W_18_O_49_ and metformin alleviate tumor hypoxia for enhanced photodynamic therapy and photothermal therapy. Acta Biomater.

[33] Jiang W, Wang L, Wang Q, Zhou H, Ma Y, Dong W, Xu H, Wang Y (2021). Reversing immunosuppression in hypoxic and immune-cold tumors with ultrathin oxygen self-supplementing polymer nanosheets under near infrared light irradiation. Adv Funct Mater.

[34] Liu X, Ye N, Liu S, Guan J, Deng Q, Zhang Z, Xiao C, Ding ZY, Zhang BX, Chen XP, Li Z, Yang X (2021). Hyperbaric oxygen boosts PD-1 antibody delivery and T cell infiltration for augmented immune responses against solid tumors. Adv Sci.

[35] McGettrick AF, O’Neill LAJ (2020). The role of HIF in immunity and inflammation. Cell Metab.

[36] Liu X, Yi X, Gu J, Ji Z, Zhu M, Shen M, Ren Y, Guo L, Liu T, Ding N, Yang K (2023). Immunoregulatory liposomes hitchhiking on neutrophils for enhanced carbon ion radiotherapy-assisted immunotherapy of glioblastoma. Nano Today.

[37] Zhang C, Jing X, Guo L, Cui C, Hou X, Zuo T, Liu J, Shi J, Liu X, Zuo X, Li J, Chang C, Fan C, Wang L (2021). Remote photothermal control of DNA origami assembly in cellular environments. Nano Lett.

[38] Zetrini AE, Lip HY, Abbasi AZ, Alradwan I, Ahmed T, He C, Henderson JT, Rauth AM, Wu XY (2023). Remodeling tumor immune microenvironment by using polymer-lipid-manganese dioxide nanoparticles with radiation therapy to boost immune response of castration-resistant prostate cancer. Research.

[39] Guo Q, Li X, Zhou W, Chu Y, Chen Q, Zhang Y, Li C, Chen H, Liu P, Zhao Z, Wang Y, Zhou Z, Luo Y, Li C, You H, Song H, Su B, Zhang T, Sun T, Jiang C (2021). Sequentially triggered bacterial outer membrane vesicles for macrophage metabolism modulation and tumor metastasis suppression. ACS Nano.

[40] Qing S, Lyu C, Zhu L, Pan C, Wang S, Li F, Wang J, Yue H, Gao X, Jia R, Wei W, Ma G (2020). Biomineralized bacterial outer membrane vesicles potentiate safe and efficient tumor microenvironment reprogramming for anticancer therapy. Adv Mater.

[41] Chen Q, Bai H, Wu W, Huang G, Li Y, Wu M, Tang G, Ping Y (2020). Bioengineering bacterial vesicle-coated polymeric nanomedicine for enhanced cancer immunotherapy and metastasis prevention. Nano Lett.

[42] Yi X, Zhou HL, Chao Y, Xiong SS, Zhong J, Chai ZF, Yang K, Liu Z (2020). Bacteria-triggered tumor-specific thrombosis to enable potent photothermal immunotherapy of cancer. Sci Adv.

[43] Huang X, Pan J, Xu F, Shao B, Wang Y, Guo X, Zhou S (2021). Bacteria-based cancer immunotherapy. Adv Sci.

[44] Lv M, Chen M, Zhang R, Zhang W, Wang C, Zhang Y, Wei X, Guan Y, Liu J, Feng K, Jing M, Wang X, Liu YC, Mei Q, Han W, Jiang Z (2020). Manganese is critical for antitumor immune responses via cGAS-STING and improves the efficacy of clinical immunotherapy. Cell Res.

[45] Ding L, Liang M, Li Y, Zeng M, Liu M, Ma W, Chen F, Li C, Reis RL, Li FR, Wang Y (2023). Zinc-organometallic framework vaccine controlled-release Zn^2+^ regulates tumor extracellular matrix degradation potentiate efficacy of immunotherapy. Adv Sci.

[46] Zhao X, Cheng H, Wang Q, Nie W, Yang Y, Yang X, Zhang K, Shi J, Liu J (2023). Regulating photosensitizer metabolism with DNAzyme-loaded nanoparticles for amplified mitochondria-targeting photodynamic immunotherapy. ACS Nano.

[47] Chen Q, Feng L, Liu J, Zhu W, Dong Z, Wu Y, Liu Z (2016). Intelligent albumin-MnO_2_ nanoparticles as pH-/H_2_O_2_-responsive dissociable nanocarriers to modulate tumor hypoxia for effective combination therapy. Adv Mater.

[48] Tian L, Chen Q, Yi X, Chen J, Liang C, Chao Y, Yang K, Liu Z (2017). Albumin-templated manganese dioxide nanoparticles for enhanced radioisotope therapy. Small.

[49] Deng Z, Xi M, Zhang C, Wu X, Li Q, Wang C, Fang H, Sun G, Zhang Y, Yang G, Liu Z (2023). Biomineralized MnO_2_ nanoplatforms mediated delivery of immune checkpoint inhibitors with STING pathway activation to potentiate cancer radio-immunotherapy. ACS Nano.

