# Supplementary material for: Differential reinforcement of cGAS-STING pathway-involved immunotherapy by biomineralized bacterial outer membrane-sensitized EBRT and RNT
Source: J Nanobiotechnology. 2024 Jun 3;22:310. doi: 10.1186/s12951-024-02565-7 (PMC11145800; doi:10.1186/s12951-024-02565-7)
Supplement: Supplementary file 1 — Additional file 1. [file 12951_2024_2565_MOESM1_ESM.doc]

**Supplementary Materials**

**Differential Reinforcement of cGAS-STING Pathway-Involved Immunotherapy by Biomineralized Bacterial Outer Membrane-Sensitized EBRT and RNT**

Mengling Shen^1^, Li Guo^1^, Hengyu Zhang^1^, Bingshu Zheng^2^, Xinpei Liu^1^, Jingyu Gu^1^, Tao Yang^3^, Chunfeng Sun^2^*, Xuan Yi^1^*

1. School of Pharmacy, Jiangsu Key Laboratory of Inflammation and Molecular Drug Targets, Nantong University, Nantong, Jiangsu, 226001, China.

2. Department of Nuclear Medicine, Affiliated Hospital of Nantong University, Nantong, Jiangsu 226001, China.

3. Department of Radiotherapy, Affiliated Hospital of Nantong University, Nantong, Jiangsu 226001, China.

* Correspondence should be sent to: xuanyi@ntu.edu.cn; sunchunfeng-nt@ntu.edu.cn.

**Experimental section**

**Materials.** DSPE-PEG_5000_ was purchased from Ruixi Biological Technology Co., Ltd. (Xi’an, China). BCA Protein Assay Kit was purchased from Beyotime Biotechnology. Manganese chloride tetrahydrate (MnCl_2_^.^4H_2_O) was purchased from Macklin. Sodium hydroxide was purchased from General-Reagent. Flow cytometric antibodies of cell surface markers were obtained from eBioscience or Biolegend. Enzyme-linked immune-sorbent assay test kits were purchased from MultiSciences Biotech Co., Ltd or Jiangsu Meimian Industrial Co., Ltd. DNA Damage Assay Kit by γ-H2AX Immunofluorescence was obtained from Beyotime Biotechnology. Calreticulin (D3E6) rabbit mAb (#12238) was obtained from Cell Signaling Technology (USA). Anti-HMGB1 antibody was obtained from abcam (ab190377, UK). TEM173/STING Rabbit Recombinant antibody was purchased from proteintech (80165-1-RR, USA). Phospho-TMEM173/STING (Ser366) antibody was purchased from affinity (AF7416, USA). Anti-beta Actin antibody was purchased from abcam (ab227387, UK). The VNP20009 strain was purchased from American Type Culture Collection (ATCC). The schematic diagrams in ToC and Figure 1a&1b&2o&4a&6a&6i had been created with BioRender.com with permission.

**The preparation of OMVs.** The VNP20009 was cultured in Luria broth (LB) medium at a 1:100 dilution and then cultured in a rotary shaker at 37 °C. After the bacterial growth status stabilized, the bacterium could be cultured in large quantities. Next, OMVs was prepared according to the published protocol.[] Briefly, 400 mL bacterial culture medium was centrifuged at 8,000 g for 20 min to remove the bacteria, followed by filtering through a 0.45 μm vacuum filter. The filtrate was then concentrated by using centrifugal filters with a molecular weight cutoff (MWCO) of 100 kDa (Millipore). The concentrated medium was filtered with 0.45 μm hydrophilic filter membrane and then centrifuged at 150,000 g for 3 h at 4 °C (Beckman Coulter). The OMV pellet was re-suspended in the phosphate buffered saline (PBS) and extruded 400 nm (21 times) and 200 nm (21 times) polycarbonate membrane in succession. Protein concentration of OMVs was determined by using BCA Protein Assay Kit.

**The development of OM@MnO_2_-PEG.** Based on the quantitative result of BCA Protein Assay Kit, OMVs containing 200 μg protein were dispersed in 2 mL deionized water, then 5 mg DSPE-PEG_5000_ was added and stirred for 6 h. Next, MnCl_2_ (0.1 mol/L, 50 μL) was slowly added into the solution system and stirred for another one hour. Then, NaOH (0.1 mol/L, 100 μL) was dropwise added under sonication, following by stirring for 2 h. Wash away the free DSPE-PEG and ions by using the centrifugal filters with MWCO of 100 kDa. The obtained material was OM@MnO_2_-PEG.

**Characterization of OM@MnO_2_-PEG.** The TEM images of OMVs and OM@MnO_2_-PEG as well as the mapping images of OM@MnO_2_-PEG were obtained by high resolution transmission electron microscopy (Thermo Fisher, FEI Talos, f200X, USA). The hydrodynamic size of OMV and OM@MnO_2_-PEG was measured with dynamic light scattering (NanoBrook, 90Plus Zeta, USA). The UV absorption of MnO_2_, OM-PEG and OM@MnO_2_-PEG was measured by UV-Vis-NIR spectrophotometer (Thermo Fisher, GENESYS 50, USA). The in vitro generation of dissolved oxygen was monitored by a portable dissolved oxygen meter (JPBJ-608, China). The concentration of Mn was tested by inductively coupled plasma mass spectrometry (ICP-MS) (PerkinElmer, Elan DRC-e, USA).

**^131^I labeling of OM@MnO_2_-PEG.** We dispersed 1mg/mL of iodogen with trichloromethane in a 2 mL EP tube and dried it by nitrogen purging. Afterward, 1 mL of OM@MnO_2_-PEG was sufficiently mixed with designated dose of ^131^I in an above-mentioned EP tube for 10 min with the vortex. Then the mixture was washed to remove free ^131^I by using the centrifugal filters with MWCO of 100 kDa. Next, a portion of the obtained ^131^I-OM@MnO_2_-PEG was placed into mouse serum. At the time points of 1, 4, 8, 16, 24 h, the deciduous ^131^I in 100 μL of ^131^I-OM@MnO_2_-PEG was collected by centrifugal filter and the radioactivity was recorded by a γ-counter (USTC Chuangxin Co., GC-400, China). By calculating the detachment rate of ^131^I, we could obtain the radioactive stability of ^131^I-OM@MnO_2_-PEG.

**Cell experiments.** 4T1 cells and RAW 264.7 cells were both cultured in a high glucose medium supplemented with 10% fetal bovine serum (FBS) and 1% penicillin/streptomycin at 37 °C under 5% CO_2_. Firstly, we evaluated the cell uptake of OM@MnO_2_-PEG by 4T1 cells and RAW 264.7 cells, respectively. The 4T1 cells or RAW 264.7 cells (2×10^5^ cells per well of 6-well plate) were separately incubated with DID-labeled OM-PEG or OM@MnO_2_-PEG with the same dose of DID for appointed time. Then, the DID fluorescence intensity of these cells was analyzed by flow cytometer (BD, BD Accuri C6, USA). Additionally, in order to test the cytotoxicity of OM-PEG and OM@MnO_2_-PEG, we pre-seeded 8×10^3^ 4T1 cells or RAW 264.7 cells per well in the 96-well plates, respectively. Then we incubated cells with various concentration of OM-PEG or OM@MnO_2_-PEG for 12 h. Finally, CCK-8 assay was used to measure the cell viability.

To check the DNA damage and observe the CRT as well as HMGB1 expression in 4T1 cells with different treatments, we firstly incubated 4T1 cells (5×10^4^ cells per well of 24-well plate) with PBS, OM-PEG, OM@MnO_2_-PEG for about 8 h and then exposed them to X-rays (6 Gy). These cells were stained with γ-H2AX (Beyotime Biotechnology, 1:50) antibody after 1 h post X-rays exposure. Cells were stained with CRT (Beyotime Biotechnology, 1:100) and HMGB1 (Abcam, 1:1000) antibody 24 h post X-rays exposure. These cells were observed by confocal microscopy (Carl Zeiss, LSM900, Germany). Furthermore, the activation of cGAS-STING pathway in 4T1 cells and RAW 264.7 cells was also checked by immunofluorescence of p-STING (Affinity, 1:100).

Next, ELISA was used to test the secretion of IFN-β. Firstly, we pre-seeded 2×10^5^ 4T1 cells in the 6-well plates and added PBS, OM-PEG or OM@MnO_2_-PEG into the corresponding groups. Specific groups of cells were exposed to X-rays (6 Gy) after about 6 hours of incubation with the materials. Then 2×10^5^ RAW 264.7 cells were added. 24 h later, the cell supernatants were collected for IFN-β detection by ELISA.

**Animals and tumor models.** Female Balb/c mice (6–8 weeks) were purchased from GemPharmatech LLC. Animal feeding and management was performed according to the SOP of Laboratory Animal Center of Nantong University. Animal experiments were performed according to the experimental protocols approved by the Laboratory Animal Center of Nantong University. 4T1 cells (2 × 10^6^) dispersed in 50 μL PBS were injected subcutaneously into the back of mouse to establish a mouse subcutaneous tumor model. When the tumor volume (volume = length × width × width/2) reached about 50 mm^3^, the mice were used for the following animal experiments.

**Blood circulation and** **bio-distribution experiments.** 4T1 tumor-bearing mice were intravenously injected with ^131^I-OM-PEG or ^131^I-OM@MnO_2_-PEG, respectively. At the time points of 0.5, 1, 2, 4, 6, 8, 10 and 24 h post injection, about 10 µL of blood was drawn from one side of orbital venous plexus. These blood samples were weighed and their radioactivity was measured by the γ-counter. 24 hour post injection, these mice were sacrificed and the major organs including heart, liver, spleen, lung, kidney and tumor were collected. These organs were weighed and their radioactivity was measured by the γ-counter. Similarly, the bio-distribution of ^131^I-OM@MnO_2_-PEG was measured at the time points of 1, 3 and 7 d post intratumoral injection.

**SPECT imaging.** For SPECT imaging, BALB/c mice bearing 4T1 tumor were intratumorally injected with ^131^I-OM@MnO_2_-PEG (about 10 μCi, 20 μL). SPECT images were obtained through a SPECT imaging system (GE, Discovery NM 630, USA) at different time points of 0, 1, 3, 5 and 7 d post injection.

**Detection of tumor hypoxia.** The tumor-bearing mice were randomly divided into five groups: PBS, OM-PEG (intravenous injection, i.v.), OM@MnO_2_-PEG (i.v.), OM-PEG (intratumoral injection, i.t.), OM@MnO_2_-PEG (i.t.). One day after indicated treatments, pimonidazole hydrochloride (60 mg/kg) was intravenously injected into the mice. Half an hour later, tumors were collected and embedded with tissue embedding medium, followed by frozen sections. Next, 4% paraformaldehyde was used to fix these frozen sections. After being washed several times and dried, the sections were blocked with FBS. Then, FITC-Mab antibody diluted with PBST-1 (PBS containing 0.1% Tween, 1% FBS, and 0.1% Triton X-100) at a ratio of 1:200. The tissue sections were incubated with this FITC-Mab for 90 min at room temperature, followed by washing several times with PBST-2 (PBS containing 0.1% Tween and 1% FBS). Finally, nuclei was stained with DAPI. After dropping anti-fluorescence quenching agent, the sections were sealed and observed by laser scanning confocal microscope.

**Tumor growth inhibition.** To observe the therapeutic effect of our treatment strategy on bilateral tumors, we firstly constructed the bilateral tumor model by subcutaneously injecting 4T1 cells (2 × 10^6^) into the right and left flanks of each mouse on the day -7 and -5, respectively. The tumor on the right flank was treated with OM-PEG, OM@MnO_2_-PEG, EBRT (Elekta, Precise, Sweden) or ^131^I-OM@MnO_2_-PEG on day 0, and the size of the tumors on the right and left flank was measured by the vernier caliper.

**Analysis of anti-tumor immunity.** To analyze the maturation of DC cells and the proportion of various T cells, lymph nodes and 4T1 tumors were firstly collected from mice with indicated treatment and then homogenized in PBS (pH 7.4) containing 1% FBS. Subsequently, sufficient cells were obtained from the tissue by cutting it into pieces and digesting it with tissue digestive enzyme that were formulated by fully dissolving collagenase I (Biosharp, CAS: 9001-12-1, BS163-100 mg), collagenase IV (Biosharp, CAS: 9001-12-1, BS165-100 mg), hyaluronidase (Biosharp, CAS: 37326-33-3, BS171-100 mg) and DNase I (Biosharp, CAS: 9003-98-9, BS137-10 mg) in 66.66 mL of complete culture medium. Next, the cells in lymph nodes were stained with FITC Anti-Mo CD11c (eBioscience, Clone: N418, 11-0114-82), APC Anti-Mo CD86 (eBioscience, Clone: GL1, 17-0862-82), PE Anti-Mo CD80 (eBioscience, Clone: 16-10A1, 12-0801-82) antibodies. The cells in tumor tissue were stained with FITC Anti-Mo CD3 (eBioscience, clone: 17A2, 11-0032-82), APC Anti-Mo CD4 (eBioscience, clone: GK1.5, 17-0041-82) and PE Anti-Mo CD8a (eBioscience, clone: 53-6.7, 12-0081-82) antibodies according to the protocol to distinguish cytotoxic T lymphocytes (CTLs, CD3^+^CD4^-^CD8^+^). The tumor cells were also stained with FITC Anti-Mo CD3 (eBioscience, clone: 17A2, 11-0032-82), APC Anti-Mo CD4 (eBioscience, clone: GK1.5, 17-0041-82) and PE Anti-Mo Foxp3 (eBioscience, clone: NRRF-30, 12-4771-82) for the analysis of the proportion of Tregs in CD4^+^ T cells. Additionally, the tumor cells were stained with Percp Anti-Mouse/Human CD11b Antibody (Biolegend, clone: M1/70, 101230), FITC Anti-Mouse F4/80 Antibody (eBioscience, clone: BM8, 11-4801-82), PE Anti-Mouse CD80 Antibody (eBioscience, clone: 16-10A1, 12-0801-82) and APC Anti-Mouse CD206 Antibody (eBioscience, clone: MR6F3, 17-2061-82) to distinguish M1 and M2 macrophages.

**Statistical analysis.** Statistical analysis was performed by using one-way analysis of variance (ANOVA) with the least significant difference post hoc test for multiple groups. P < 0.05 was considered statistically significant.

**References**

[1] Qing, S.; Lyu, C.; Zhu, L.; Pan, C.; Wang, S.; Li, F.; Wang, J.; Yue, H.; Gao, X.; Jia, R.; Wei, W.; Ma, G., Biomineralized bacterial outer membrane vesicles potentiate safe and efficient tumor microenvironment reprogramming for anticancer therapy. Adv. Mater. 2020;32:2002085.

[2] Chen, Q.; Bai, H.; Wu, W.; Huang, G.; Li, Y.; Wu, M.; Tang, G.; Ping, Y., Bioengineering bacterial vesicle-coated polymeric nanomedicine for enhanced cancer immunotherapy and metastasis prevention. Nano Lett. 2020;20:11-21.

**
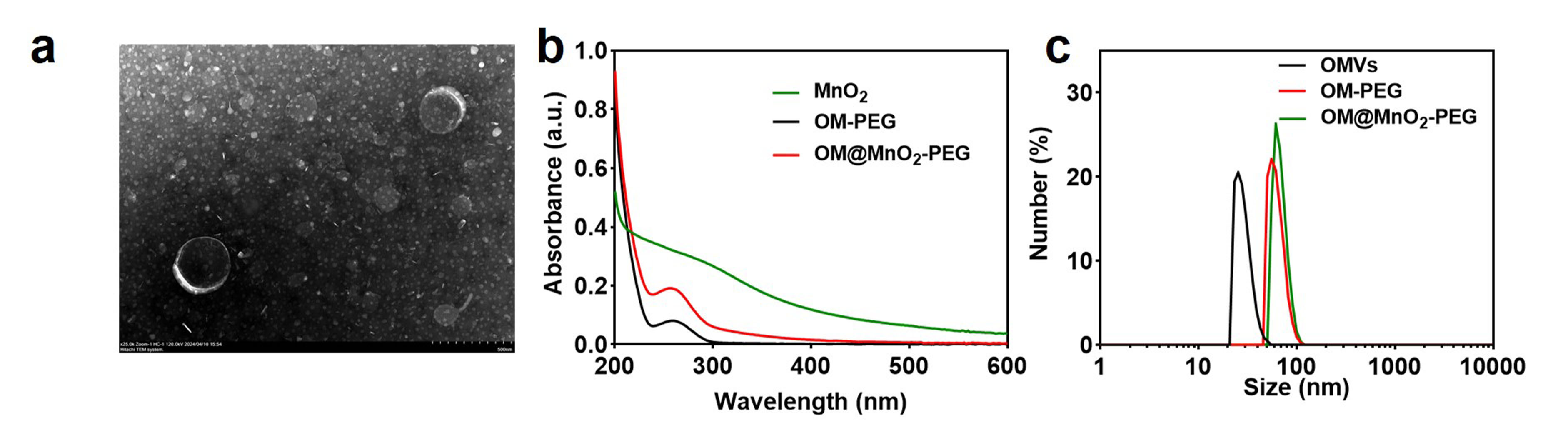
**

**Figure S1:** (a) TEM of OMVs. (b) UV-Vis-NIR absorption spectra of MnO_2_, OM-PEG and OM@MnO_2_-PEG. (c) Hydrodynamic size distribution of OMVs, OM-PEG and OM@MnO_2_-PEG.


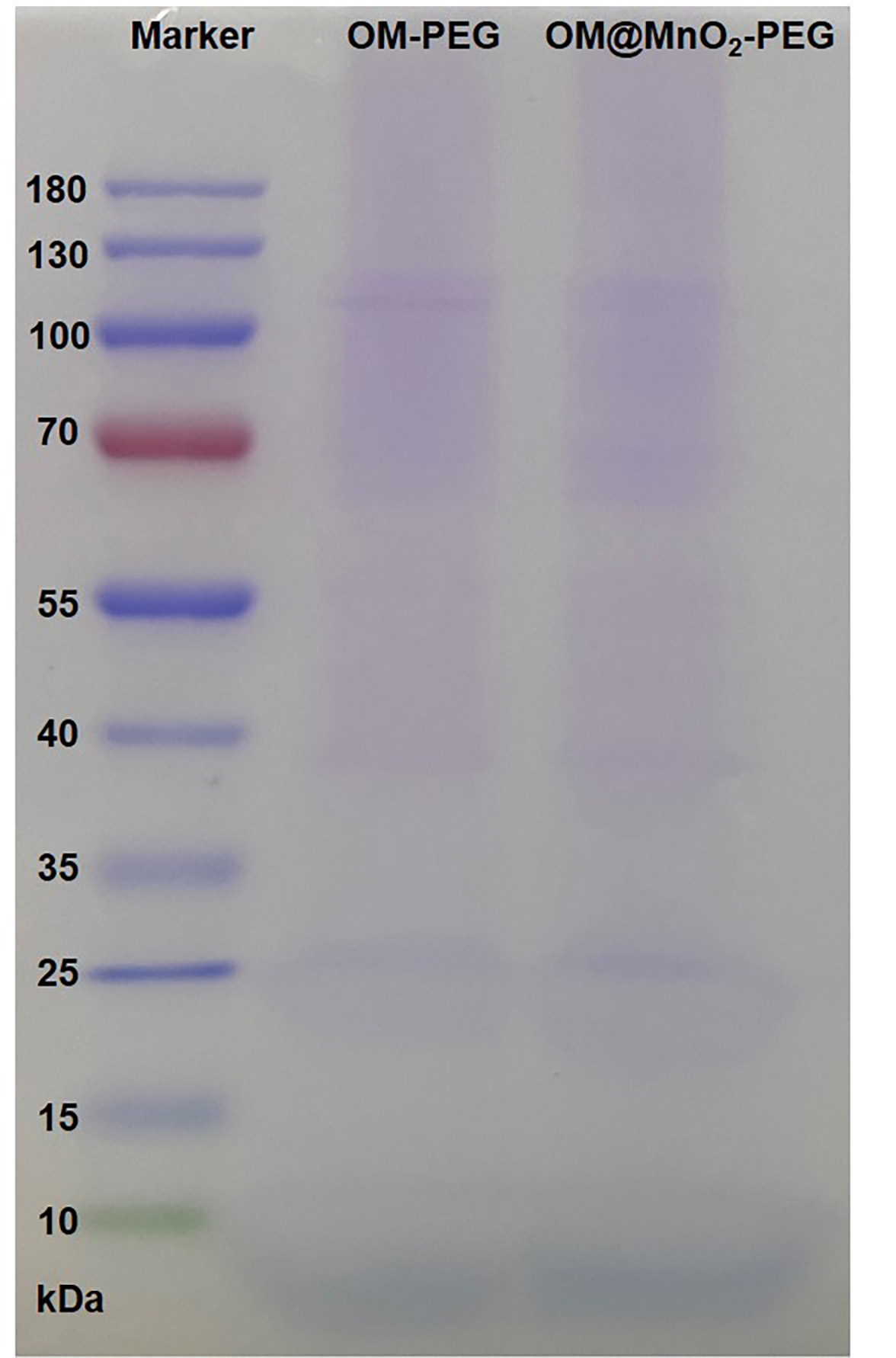


**Figure S2:** The SDS-PAGE protein analysis of OM-PEG and OM@MnO_2_-PEG.

**
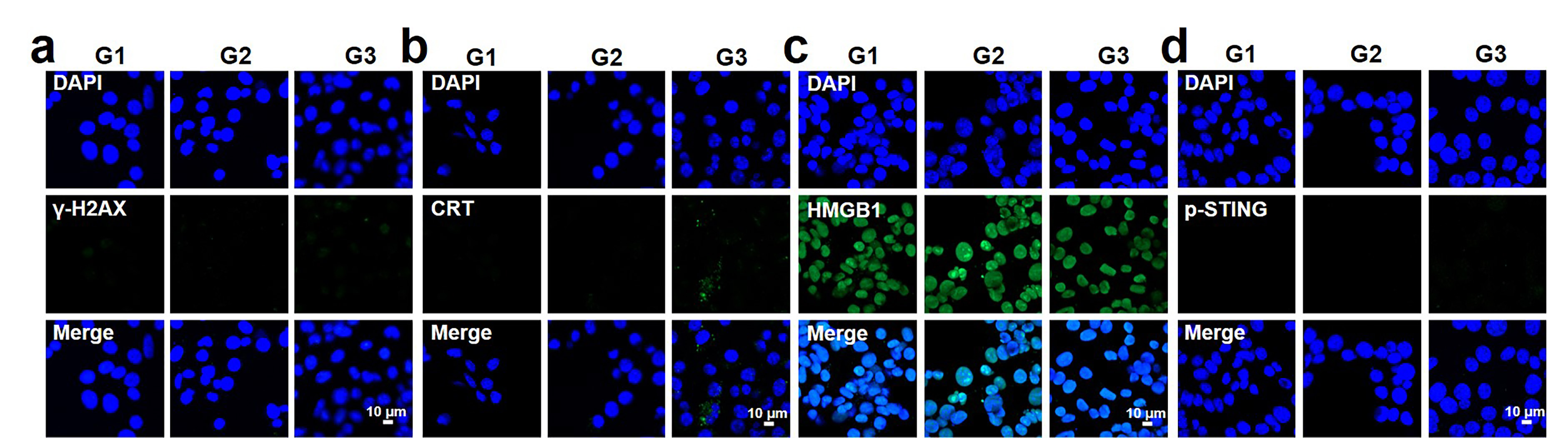
**

**Figure S3:** Fluorescent images of γ-H2AX (a), CRT (b) HMGB1 (c) and p-STING (d) expression in 4T1 cells with the indicated treatments (scale bar: 10 μm). G1: PBS; G2: OM-PEG; G3: OM@MnO_2_-PEG.


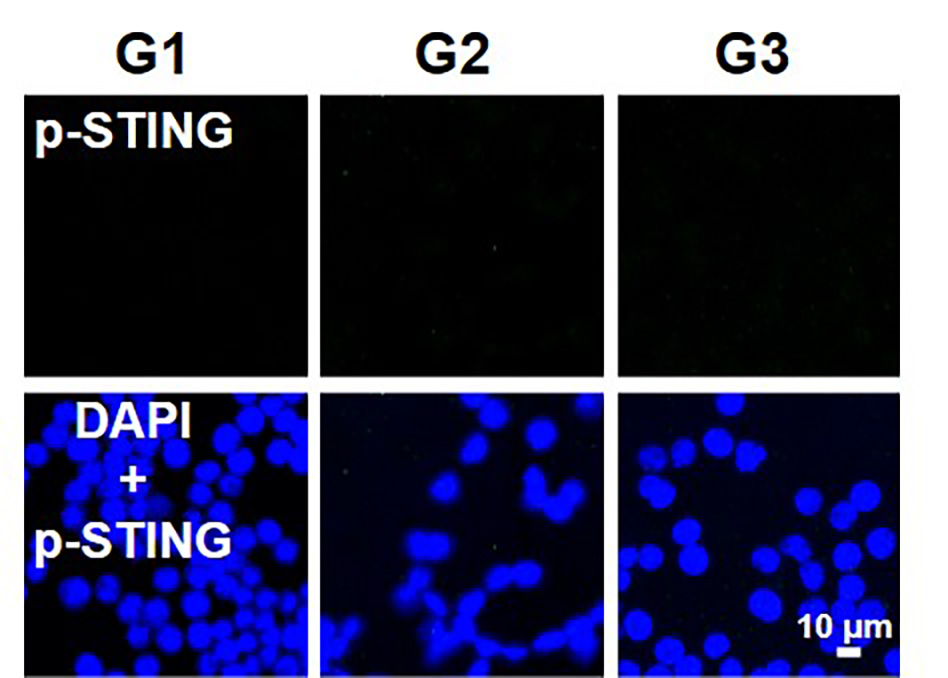


**Figure S4:** Fluorescent images of p-STING expression in RAW 264.7 cells with the indicated treatments (scale bar: 10 μm). G1: PBS; G2: OM-PEG; G3: OM@MnO_2_-PEG.


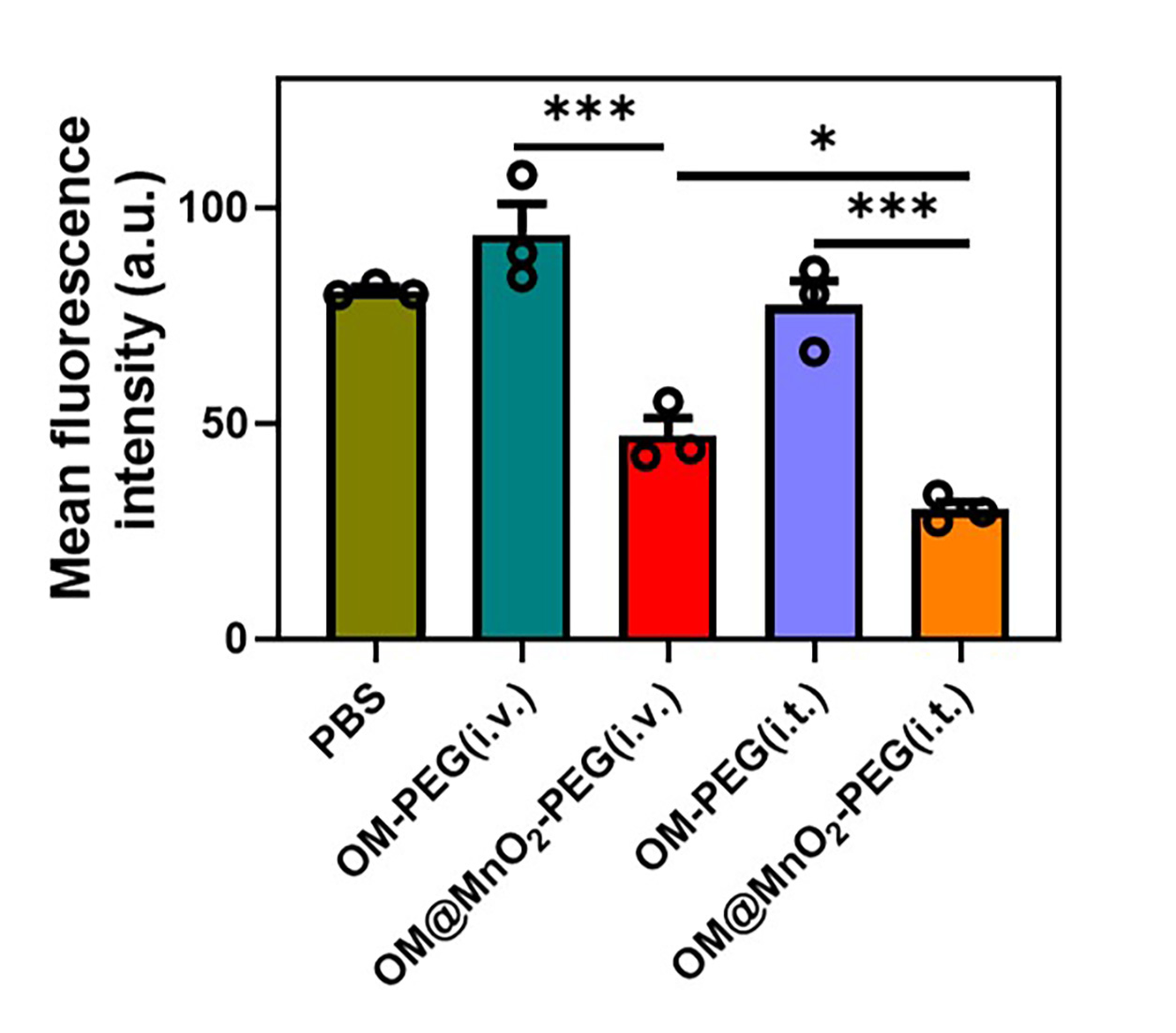


**Figure S5:** Corresponding semi-quantitative analysis of hypoxia fluorescence intensity in primary tumors with the indicated treatments (n=3). Data are presented as Mean ± SEM. Statistical significance was analyzed by one-way analysis of variance (ANOVA) with the least significant difference post hoc test. (*P < 0.05, **P < 0.01, ***P < 0.001 and ****P < 0.0001).


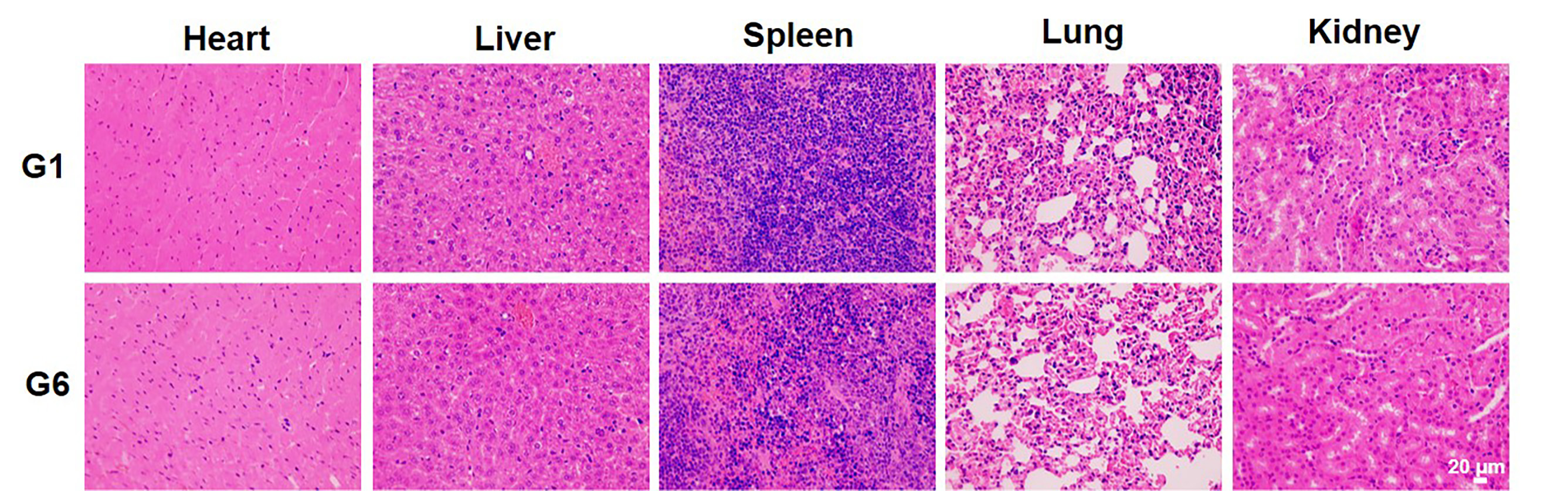


**Figure S6:** H&E staining images of organs including heart, liver, spleen, lung and kidney collected from mice treated with PBS or OM@MnO_2_-PEG + X-rays three days post treatment. G1: PBS; G6: OM@MnO_2_-PEG + X-rays.


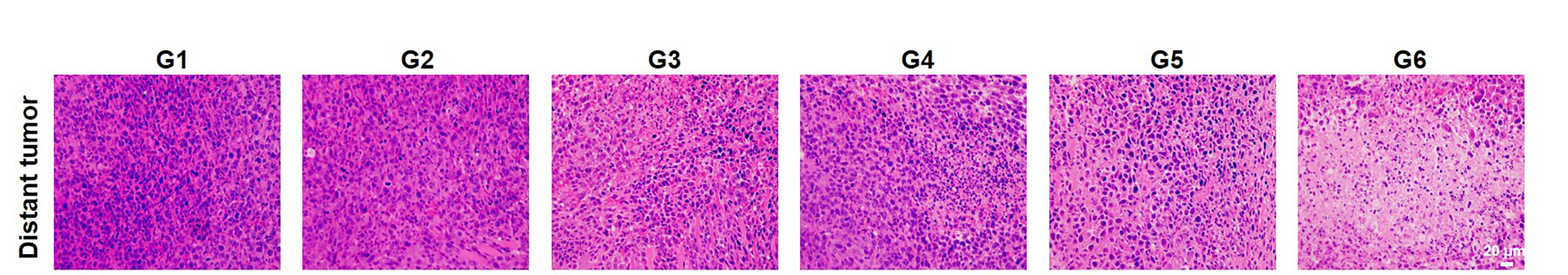


**Figure S7:** H&E staining of distant tumor sections with various treatments (scale bar: 20 μm). G1: PBS; G2: OM-PEG; G3: OM@MnO_2_-PEG; G4: X-rays; G5: OM-PEG + X-rays; G6: OM@MnO_2_-PEG + X-rays.


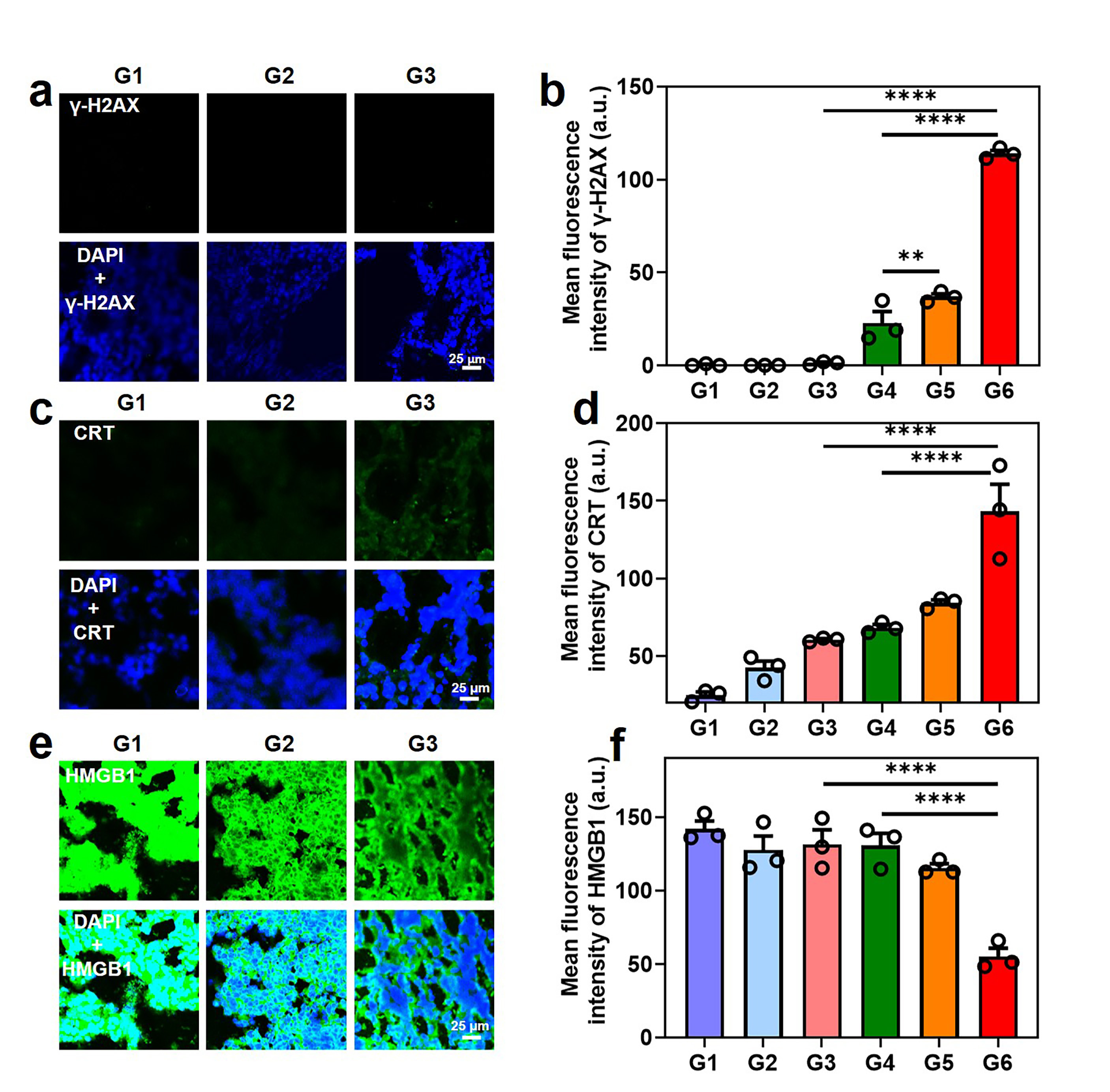


**Figure S8:** Fluorescent images and corresponding semi-quantitative analysis (n=3) of γ-H2AX (a&b), CRT (c&d) and HMGB1 (e&f) expression in primary tumor sections with the indicated treatments. G1: PBS; G2: OM-PEG; G3: OM@MnO_2_-PEG; G4: X-rays; G5: OM-PEG + X-rays; G6: OM@MnO_2_-PEG + X-rays. Data are presented as Mean ± SEM. Statistical significance was analyzed by one-way analysis of variance (ANOVA) with the least significant difference post hoc test. (*P < 0.05, **P < 0.01, ***P < 0.001 and ****P < 0.0001).


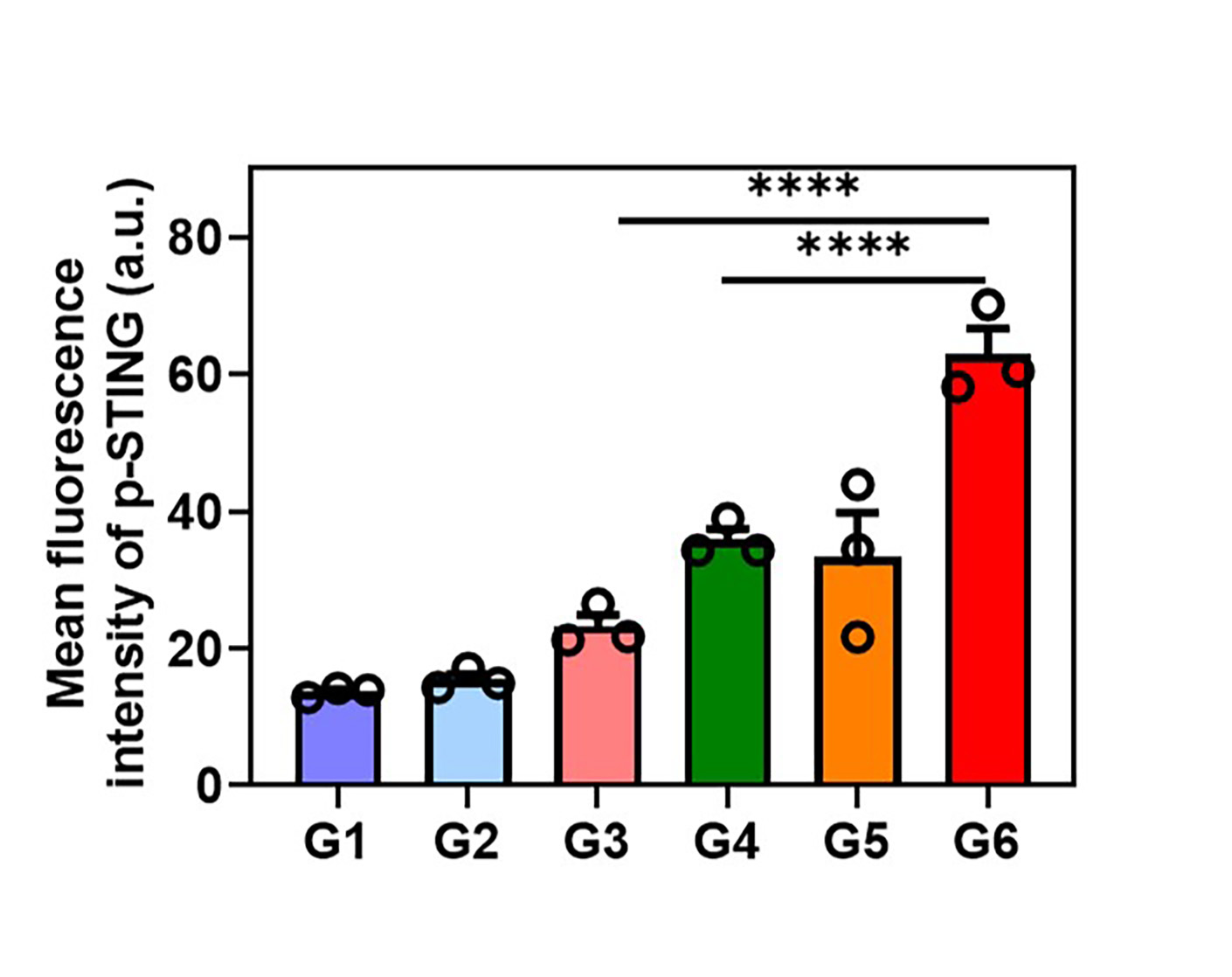


**Figure S9:** Corresponding semi-quantitative analysis of p-STING expression in primary tumor sections with the indicated treatments (n=3). G1: PBS; G2: OM-PEG; G3: OM@MnO_2_-PEG; G4: X-rays; G5: OM-PEG + X-rays; G6: OM@MnO_2_-PEG + X-rays. Data are presented as Mean ± SEM. Statistical significance was analyzed by one-way analysis of variance (ANOVA) with the least significant difference post hoc test. (*P < 0.05, **P < 0.01, ***P < 0.001 and ****P < 0.0001).


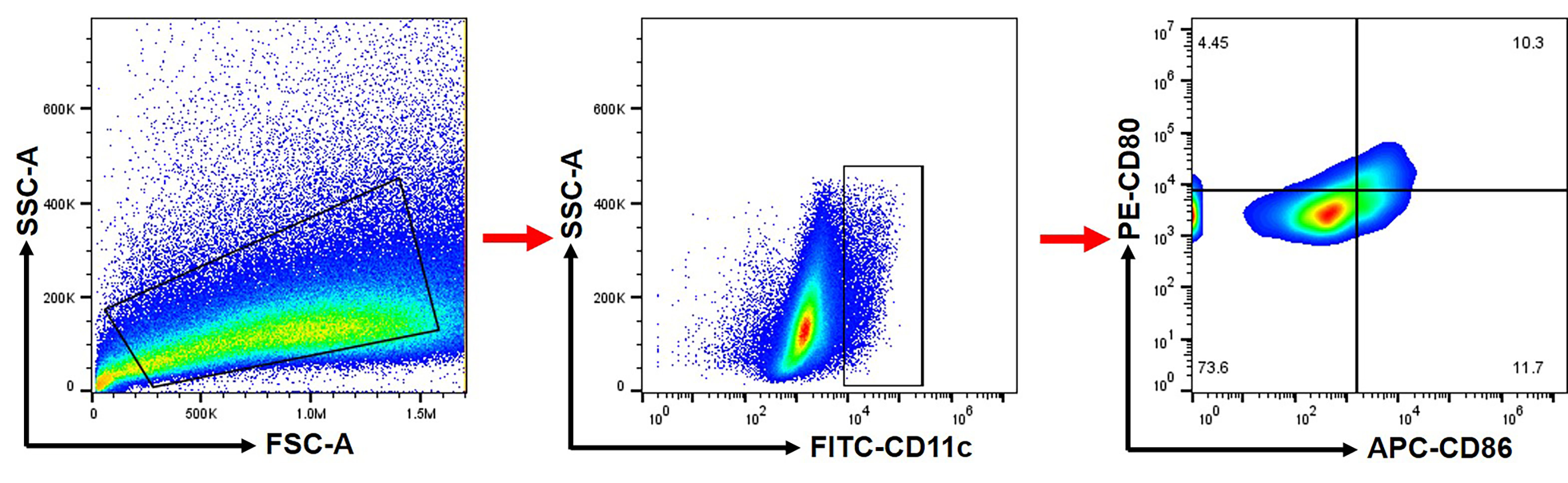


Figure S10: Gating strategy of flow cytometry for detecting DC maturation.


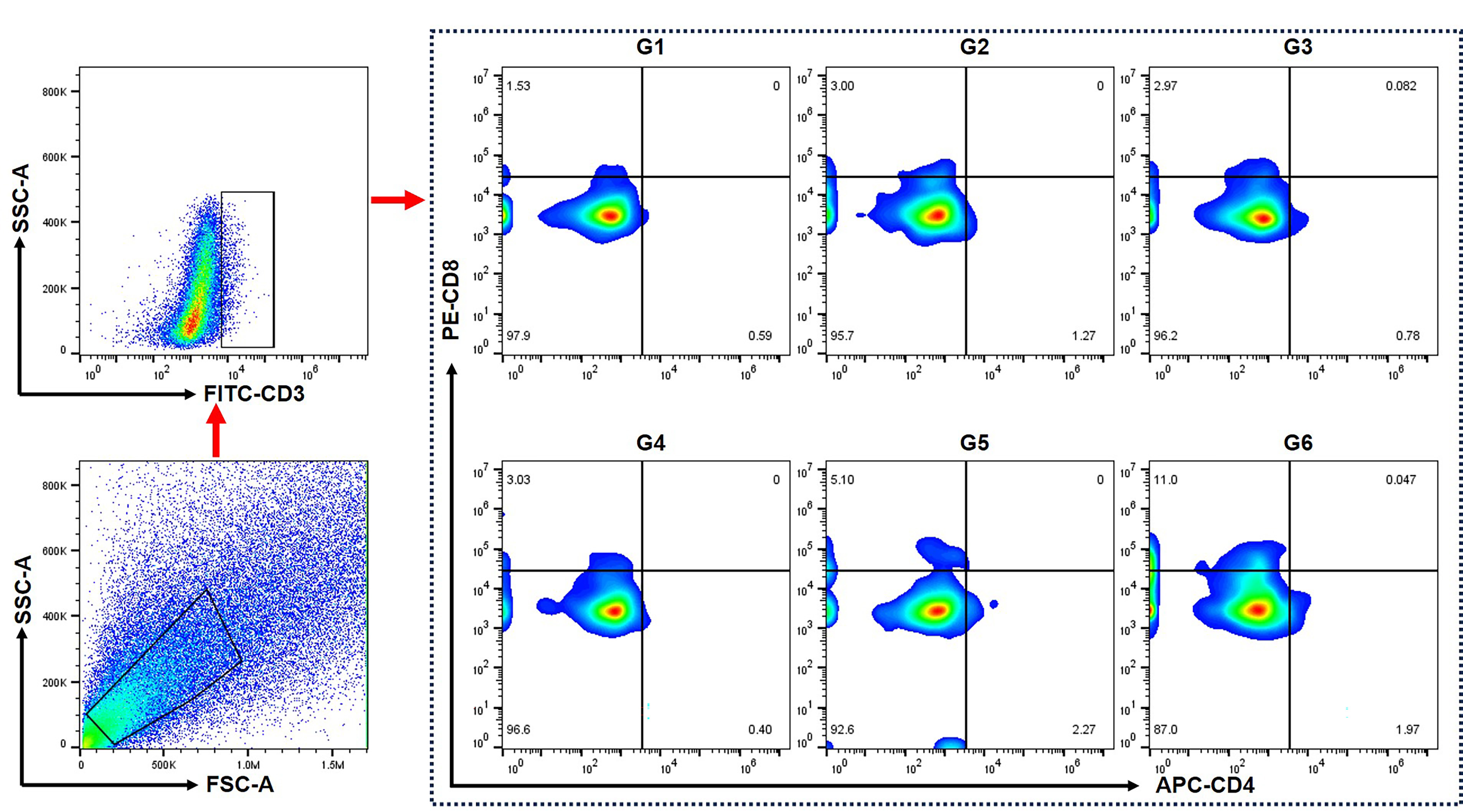


**Figure S11:** Gating strategy of flow cytometry and representative flow cytometry plots of tumor-infiltrating CD8^+^ T cells among CD3^+^ cells in primary tumors with different treatments at day 3 post injection. G1: PBS; G2: OM-PEG; G3: OM@MnO_2_-PEG; G4: X-rays; G5: OM-PEG + X-rays; G6: OM@MnO_2_-PEG + X-rays.


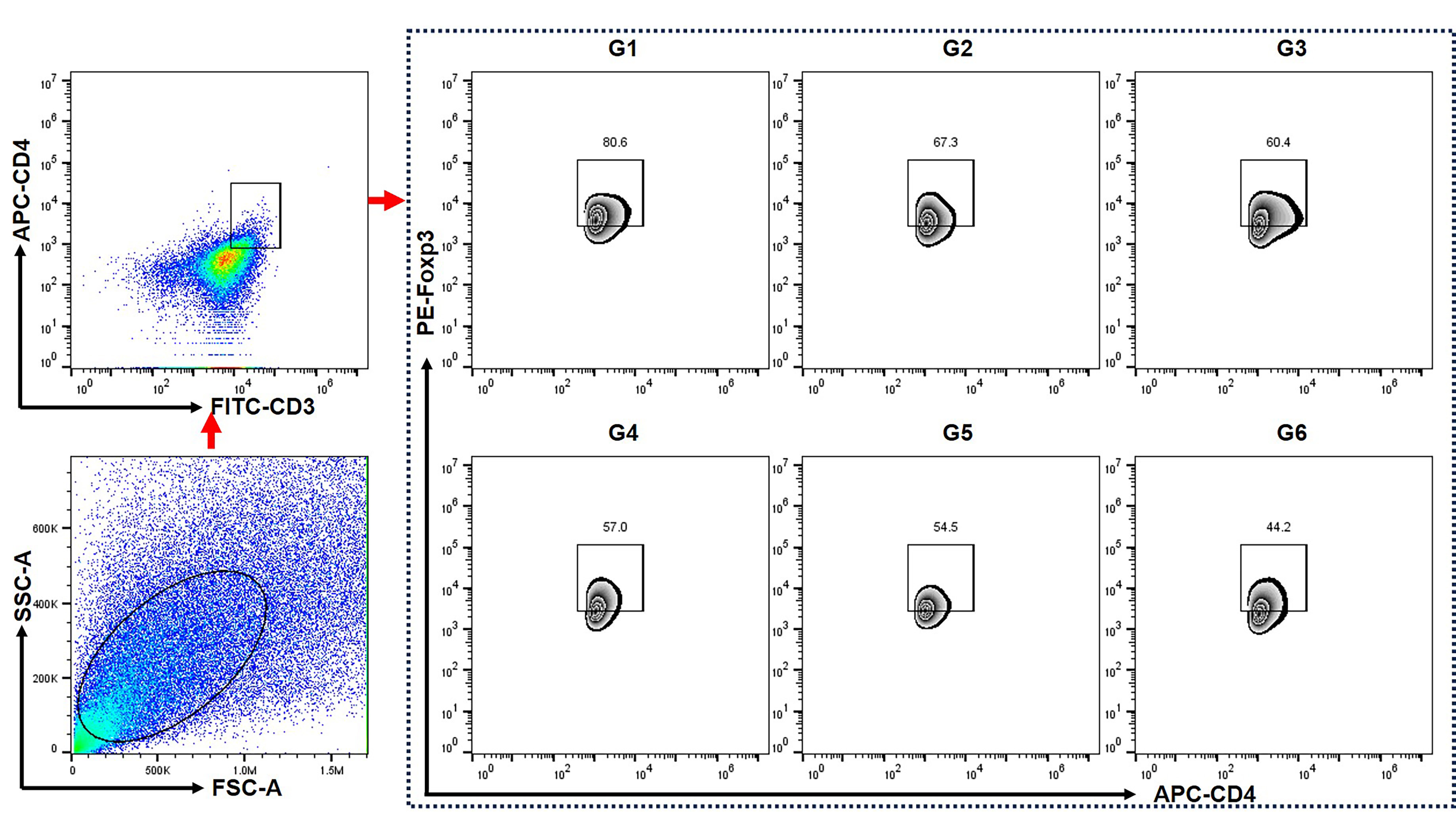


**Figure S12:** Gating strategy of flow cytometry and representative flow cytometry plots of Treg cells gated on CD3^+^CD4^+^ T cells in primary tumors at day 3 post injection. G1: PBS; G2: OM-PEG; G3: OM@MnO_2_-PEG; G4: X-rays; G5: OM-PEG + X-rays; G6: OM@MnO_2_-PEG + X-rays.


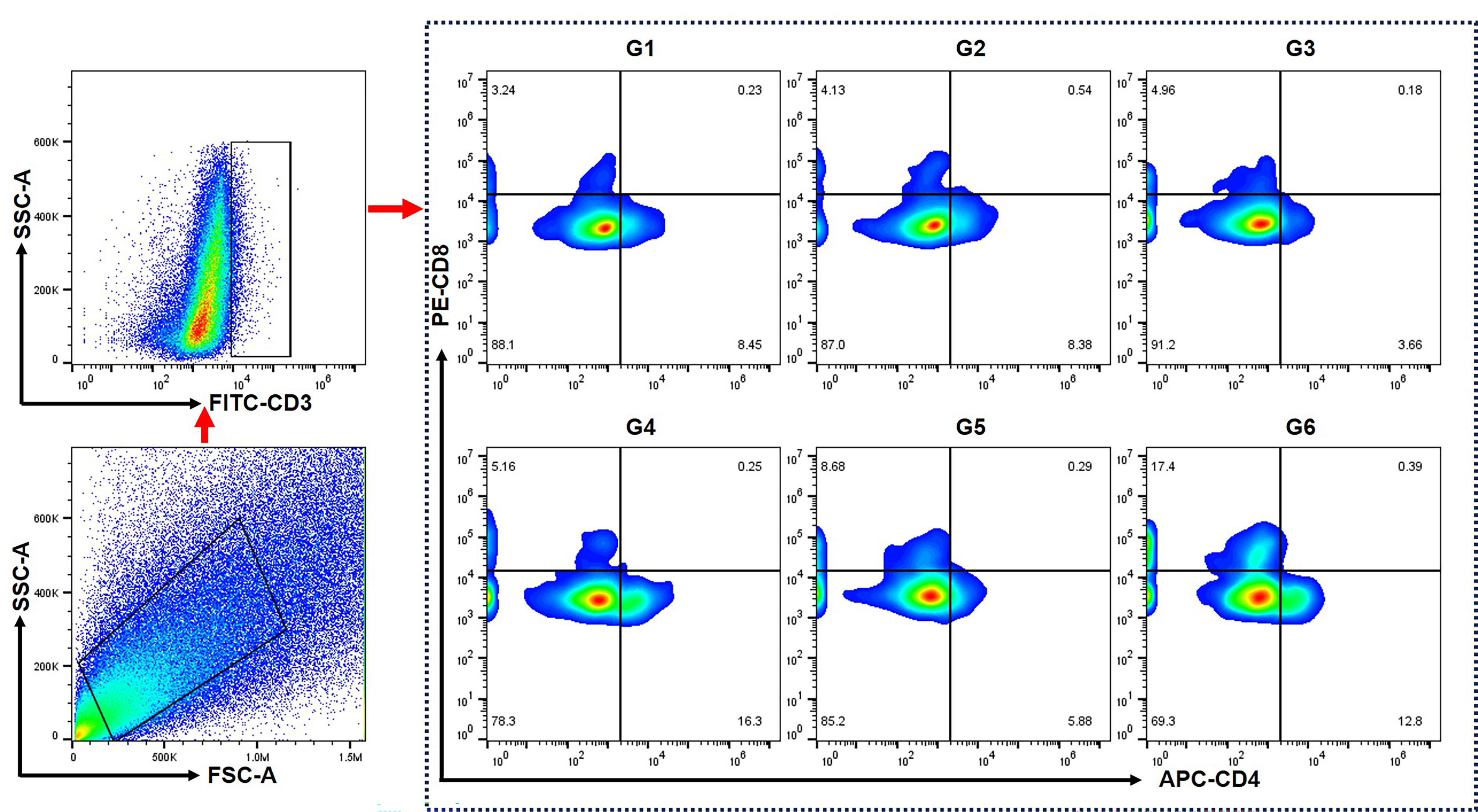


**Figure S13:** Gating strategy of flow cytometry and representative flow cytometry plots of tumor-infiltrating CD8^+^ T cells among CD3^+^ cells in distant tumors with different treatments at day 3 post injection. G1: PBS; G2: OM-PEG; G3: OM@MnO_2_-PEG; G4: X-rays; G5: OM-PEG + X-rays; G6: OM@MnO_2_-PEG + X-rays.


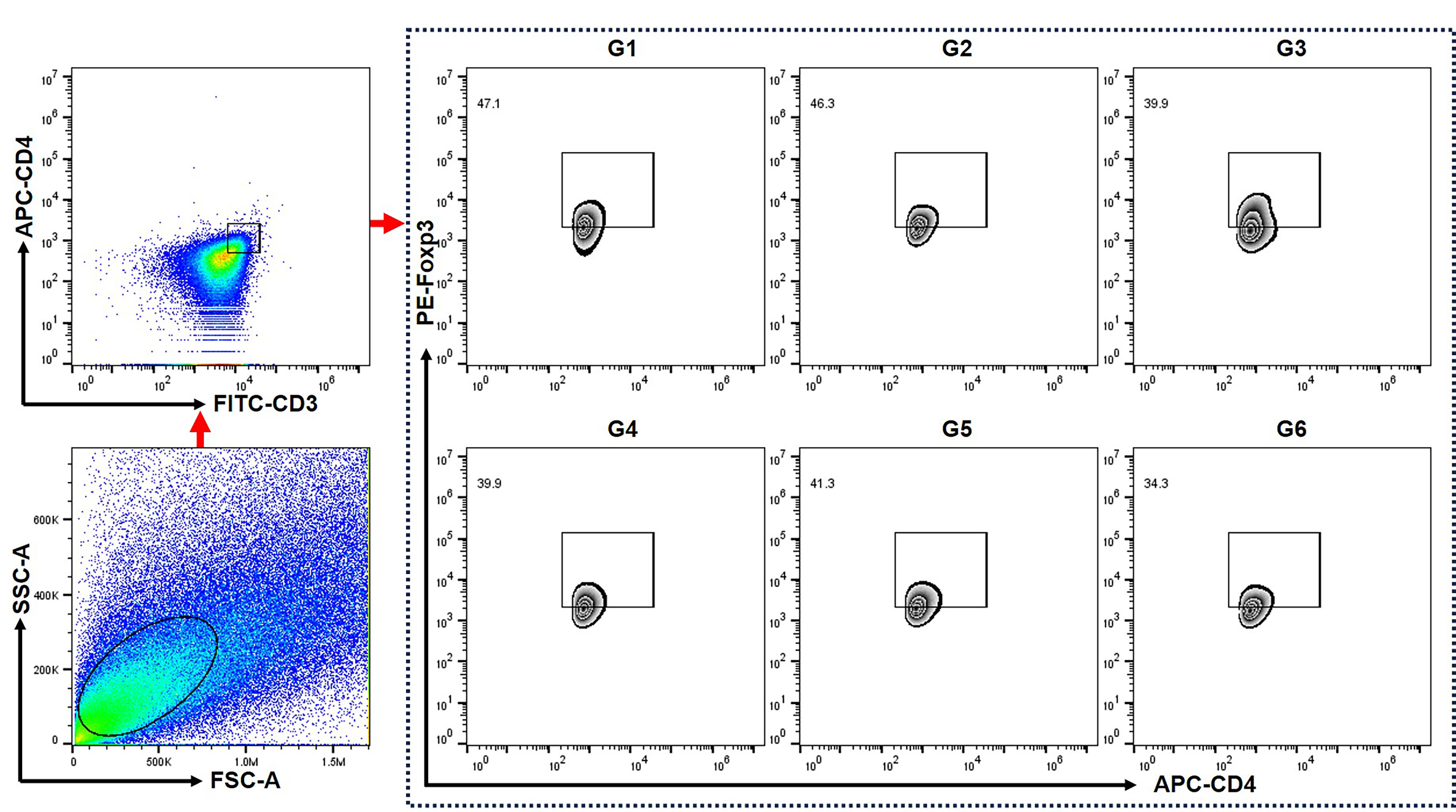


**Figure S14:** Gating strategy of flow cytometry and representative flow cytometry plots of Treg cells gated on CD3^+^CD4^+^ T cells in distant tumors at day 3 post injection. G1: PBS; G2: OM-PEG; G3: OM@MnO_2_-PEG; G4: X-rays; G5: OM-PEG + X-rays; G6: OM@MnO_2_-PEG + X-rays.

**
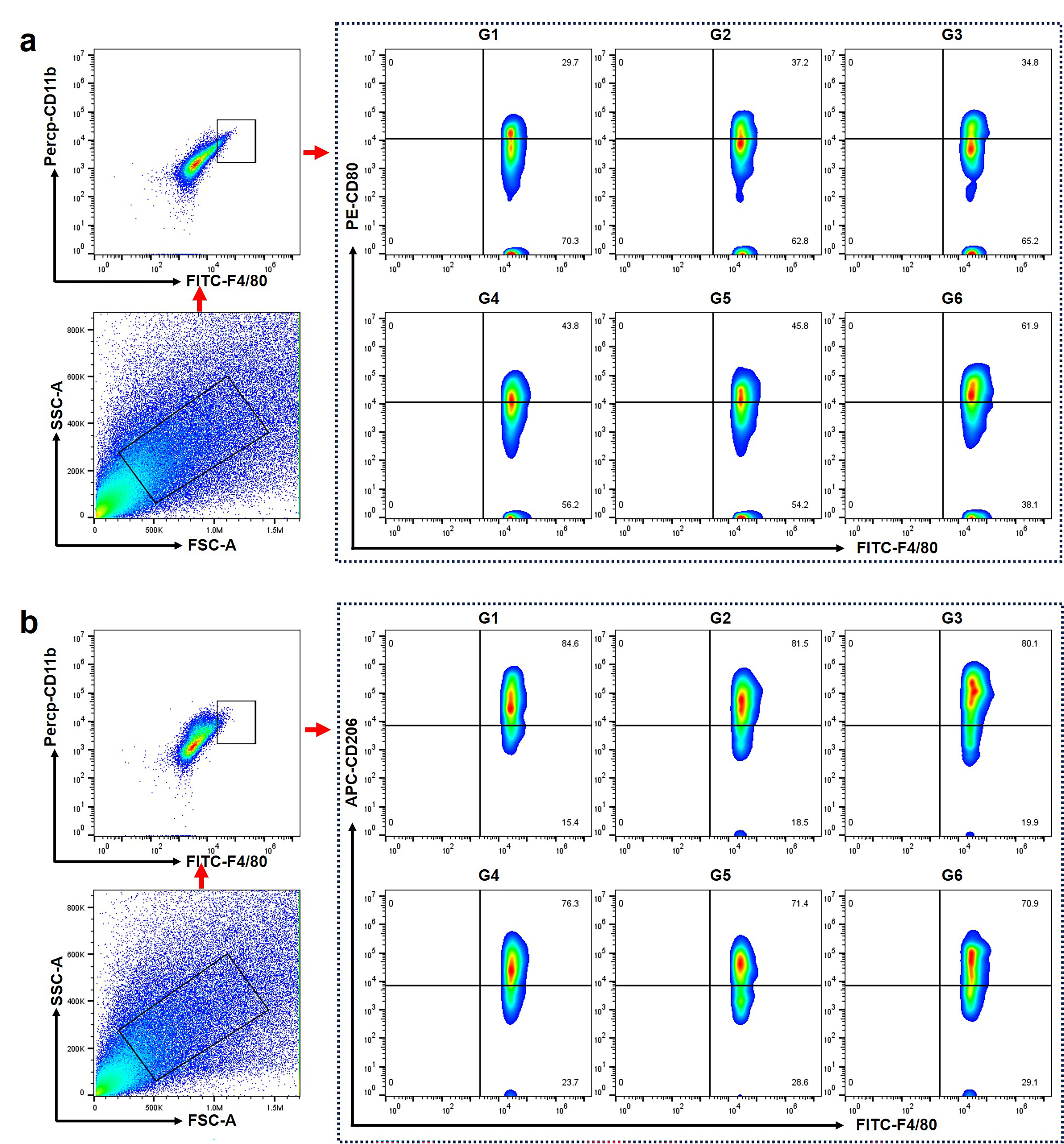
**

**Figure S15:** (a&b) Gating strategy of flow cytometry and the representative flow cytometry profiles of M1 macrophages (CD11b^+^F4/80^+^CD80^+^) and M2 macrophages (CD11b^+^F4/80^+^CD206^+^) in primary tumors at day 3 after different treatments. G1: PBS; G2: OM-PEG; G3: OM@MnO_2_-PEG; G4: X-rays; G5: OM-PEG + X-rays; G6: OM@MnO_2_-PEG + X-rays.


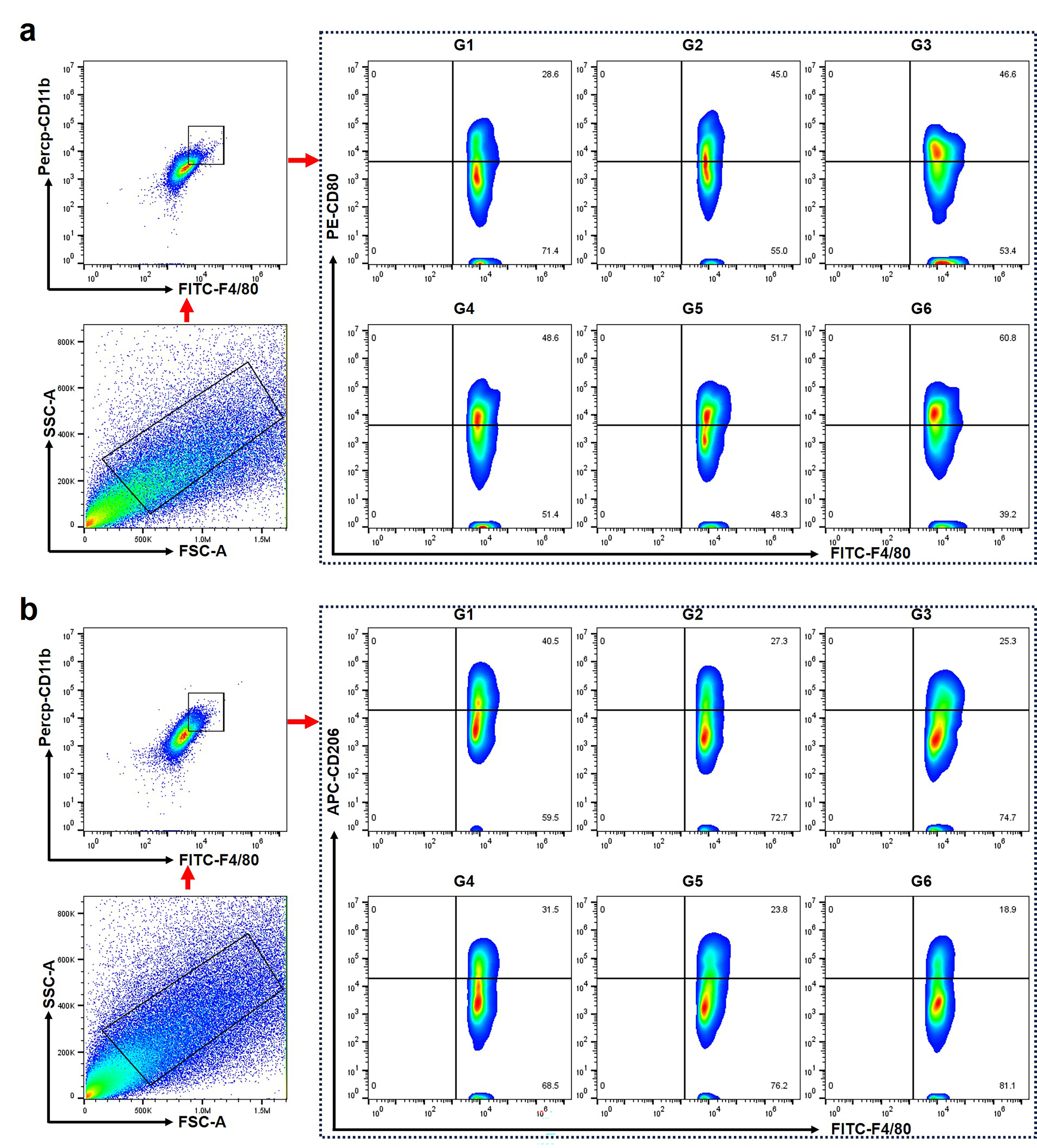


**Figure S16:** (a&b) Gating strategy of flow cytometry and the representative flow cytometry profiles of M1 macrophages (CD11b^+^F4/80^+^CD80^+^) and M2 macrophages (CD11b^+^F4/80^+^CD206^+^) in distant tumors at day 3 after different treatments. G1: PBS; G2: OM-PEG; G3: OM@MnO_2_-PEG; G4: X-rays; G5: OM-PEG + X-rays; G6: OM@MnO_2_-PEG + X-rays.

**-PEG**

**@MnO_2_-PEG**

**Marker**

**kDa**

**i**
